# Analysis of Epithelial and Mesenchymal Markers in Ovarian Cancer Reveals Phenotypic Heterogeneity and Plasticity

**DOI:** 10.1371/journal.pone.0016186

**Published:** 2011-01-14

**Authors:** Robert Strauss, Zong-Yi Li, Ying Liu, Ines Beyer, Jonas Persson, Pavel Sova, Thomas Möller, Sari Pesonen, Akseli Hemminki, Petra Hamerlik, Charles Drescher, Nicole Urban, Jiri Bartek, André Lieber

**Affiliations:** 1 Division of Medical Genetics, University of Washington, Seattle, Washington, United States of America; 2 Department of Pathology, University of Washington, Seattle, Washington, United States of America; 3 Department of Neurology, University of Washington, Seattle, Washington, United States of America; 4 Cancer Gene Therapy Group, University of Helsinki and Helsinki University Central Hospital, Helsinki, Finland; 5 Fred Hutchinson Cancer Research Center, Seattle, Washington, United States of America; 6 Danish Cancer Society, Department of Cell Cycle and Cancer, Center for Genotoxic Stress Research, Copenhagen, Denmark; 7 Laboratory of Genomic Integrity and Institute of Molecular and Translational Medicine, Palacky University, Olomouc, Czech Republic; University of Bergen, Norway

## Abstract

In our studies of ovarian cancer cells we have identified subpopulations of cells that are in a transitory E/M hybrid stage, i.e. cells that simultaneously express epithelial and mesenchymal markers. E/M cells are not homogenous but, *in vitro* and *in vivo*, contain subsets that can be distinguished based on a number of phenotypic features, including the subcellular localization of E-cadherin, and the expression levels of Tie2, CD133, and CD44. A cellular subset (E/M-MP) (membrane E-cadherin^low^/cytoplasmic E-cadherin^high^/CD133^high^, CD44^high^, Tie2^low^) is highly enriched for tumor-forming cells and displays features which are generally associated with cancer stem cells. Our data suggest that E/M-MP cells are able to differentiate into different lineages under certain conditions, and have the capacity for self-renewal, i.e. to maintain a subset of undifferentiated E/M-MP cells during differentiation. Trans-differentiation of E/M-MP cells into mesenchymal or epithelial cells is associated with a loss of stem cell markers and tumorigenicity. *In vivo* xenograft tumor growth is driven by E/M-MP cells, which give rise to epithelial ovarian cancer cells. In contrast, *in vitro*, we found that E/M-MP cells differentiate into mesenchymal cells, in a process that involves pathways associated with an epithelial-to-mesenchymal transition. We also detected phenotypic plasticity that was dependent on external factors such as stress created by starvation or contact with either epithelial or mesenchymal cells in co-cultures. Our study provides a better understanding of the phenotypic complexity of ovarian cancer and has implications for ovarian cancer therapy.

## Introduction

It has been suggested that tumor re-growth, as well as chemotherapy resistance and metastasis, are dependent on a small sub-population of cancer cells within the tumor that are thought to represent cancer stem cells (CSCs). A defining hallmark of stem cells, in both normal and malignant tissue, is the ability to self-renew but simultaneously give rise to daughter cells that are committed to differentiation into phenotypes that often cross lineages. To achieve this, stem cells can undergo an asymmetric cell division whereby they segregate cell fate determinants into only one of the two daughter cells [1]. In adult mammals, stem cells have been characterized for a number of tissues, including the blood system, central nervous system, muscle, colon, breast, and bone/cartilage.

For cancer stem cells in solid tumors, much can be learned from studies of hematopoietic stem cells (HSC) for which it has been shown that the transplantation of a single cell into a myeloablated recipient can reconstitute the entire blood system. This definitive HSC gives rise to a hierarchy of pluripotent progenitors that become progressively restricted in their differentiation potential. Leukemia is thought to originate either from HSCs that acquired genetic or epigenetic changes and became partially differentiated and tumorigenic, or from progenitors that acquired the capacity to self-renew [2]. The existence of stem cells for certain types of leukemia is strongly supported by lentiviral tagging of human acute myelogenous leukemia cells and the observation of individual clones present in NOD–SCID mice after serial transplantation of the tagged cells [3]. Studies of leukemia stem cells also indicated great phenotypic plasticity depending on the stage of tumor growth, tumor microenvironment, and external factors such as stress created by radio-or chemotherapy [2].

The presence of CSCs in solid tumors has been proposed for human cancers including breast [4], brain [5], colon [6], head and neck [7], pancreatic [8], prostate [9], ovarian [10], [11], [12], and skin cancer [13]. Solid tumor stem cells have been defined as “a small subset of cancer cells within a cancer that constitute a reservoir of self-sustaining cells with the exclusive ability to self-renew and to cause the heterogeneous lineages of cancer cells that comprise the tumor” [14]. Several cell surface markers, including CD24, CD44, CD133, CD166, EpCAM, or dye efflux assays have been used to sort populations of putative cancer stem cells from primary tumor cultures or cell suspensions obtained from tumor biopsies. After transplantation into immunodeficient mice, tumors form from several hundred marker-positive cells, whereas for marker-negative cells orders of magnitudes higher numbers are needed to achieve the same frequency of tumor formation (for a review: see [15]. Recently, using an improved xenotransplantation technique, a study with human melanoma cells showed tumor formation after inoculation of one tumor cell [16] and thereby an important step towards the proof of CSC existence was made.

For most carcinomas, progression toward malignancy is accompanied by loss of epithelial differentiation and a shift towards a mesenchymal phenotype (EMT) [17]. EMT exacerbates motility and invasiveness of many cell types and is often considered a prerequisite for tumor infiltration and metastasis. EMT is characterized by increased expression of mesenchymal markers (vimentin, thrombospondin, N-cadherin, vitronectin), increased expression of extracellular matrix compounds (collagen IV and fibronectin), decreased expression of epithelial markers (E-cadherin, Occludin, Desmoplakin, and Mucin1), altered location of transcription factors (β-catenin, Snail, Slug, Twist, Sox 10, and NFκB) and activation of kinases (ERK1, ERK2, and PI3K/AKT) [17]. There are also numerous examples of advanced carcinomas showing that mesenchymal cells can regain characteristics of epithelial cells, a process called mesenchymal to epithelial transition (MET) [18]. Apparently, the epithelial phenotype of cancer cells and the ability to form physical barriers represent a mechanism that restricts access of drugs, antibodies, or immune cells to the sites of tumors. It has been speculated that both EMT and MET involve cells in a metastable hybrid state, e.g. cells with features of both epithelial and mesenchymal cells [19].

Our studies have focused on ovarian cancer. Ovarian cancer is the fourth most common cancer in women and has the highest mortality rate of all cancers of the female reproductive system. Approximately 90% of human ovarian cancer arises from the ovarian surface epithelium (OSE). The mesodermally derived normal OSE shows epithelial and mesenchymal features, characterized by the expression of both keratin and vimentin [20]. Interestingly, only low levels of E-cadherin are prominent in OSE cells [21] and its expression is restricted to inclusion cysts and deep clefts, remarkably to areas where early malignant events are believed to occur [22]. The integrity of OSE layers is primarily maintained by N-cadherin, which further highlights the epithelial/mesenchymal phenotype in this tissue [23], [24]. It is thought that OSE cells adapt to changes in the cellular microenvironment by transitions between epithelial and mesenchymal stages [20]. Such abilities are usually restricted to immature, regenerating, or neoplastic epithelia and therefore render a unique phenotypic plasticity. It is thought that this plasticity lies at the origin of ovarian cancer. Notably, ovarian carcinomas show a unique feature when compared to other epithelial derived cancers. Whereas the latter are characterized by the loss of epithelial properties during tumor progression, elevated expression of E-cadherin is observed for primary neoplastic ovarian epithelia [25]. However, this initial shift towards a more differentiated phenotype early in tumor progression appears then to be followed by a reacquisition of mesenchymal features in more advanced ovarian tumors, involving a secondary loss of E-cadherin [26], [27], [28]. Interestingly, the epithelial/mesenchymal phenotype apparent in OSE cells [29] is also found in the invasive front of colon [30] and breast cancer [31], and in normal epidermal tissue during wound repair [32].

In this study, we provide supporting data for one of the key features of CSC, by demonstrating pluripotency, i.e. the ability to give rise to phenotypically heterogeneous daughter cells. Our data suggest that in ovarian cancer cells these features are intrinsically linked with the phenomena of EMT and MET.

## Results

### Primary ovarian cancer cultures contain putative CSCs

We established 51 ovarian cancer cultures from biopsies/ascites of grade III and IV carcinomas (Table S1). After digestion of tumor biopsies with collagenase and trypsin, cell suspensions were cultured in Mammary Epithelial Basal Medium supplemented with EGF, insulin, hydrocortisone, bovine pituitary extract (MEGM) and 1–2% FBS for up to 5 days. In order to confirm their tumorigenic potential and to eliminate fibroblasts and leukocytes, primary cells were injected into the mammary fat pad of immunodeficient SCID-beige (C.B-Igh-1b/GbmsTac-Prkdcscid-Lystbg N7) mice. Nine primary cultures formed xenograft tumors after transplantation of 1×10^6^ cells (see Table S1). Xenografts were then excised, digested with proteases and cultured in MEGM with 10% FBS. For one culture, secondary and tertiary xenografting was performed. Sections and suspensions of original biopsies were named ovc-biopsy, ovc805-biopsy, etc; primary cultures derived from patient biopsies were named ovc316-PC, ovc805-PC, etc; sections or tumor suspensions from xenograft tumors derived from primary cultures were named ovc316-X, ovc805-X, etc; cultures derived from xenografts were named ovc316-XC, ovc805-XC, etc.

**Figure 1:**
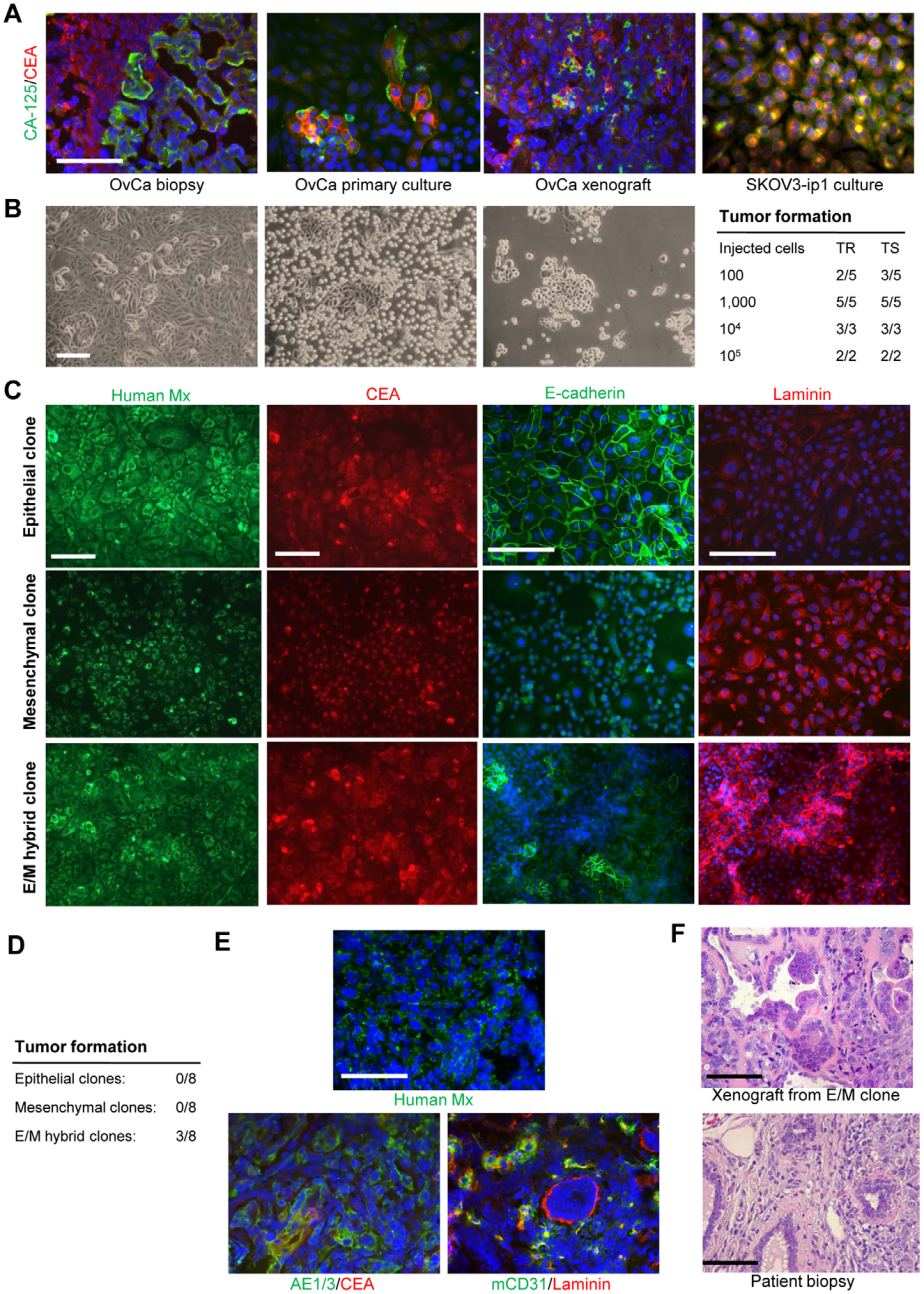
Primary ovarian cancer cultures are heterogeneous and contain putative CSCs. **A**) Tumor biopsies and primary cultures stain positive for ovarian cancer markers CA-125 (green) and CEA (red) and show higher heterogeneity than the established cell line SKOV3-ip1. Shown are ovc316-biospy, ovc316-PC, and ovc316-X. Analysis of ovc1208-biopsy, ovc1208-PC, ovc0117-biopsy, ovc0117-PC, ovc100506-biopsy, and ovc100506-PC revealed the same heterogeneity. **B**) Light microscopy of ovarian cancer culture ovc316-XC. The culture contains two distinct populations with regards to trypsin sensitivity. Left panel: an early passage culture untreated (left), after 10 min trypsin treatment (center), and after trypsin-sensitive cells were removed (right). Right panel: Tumor formation after transplantation of trypsin-resistant (TR) and trypsin-sensitive (TS) cells into SCID-beige mice. Tumor formation was evaluated 3 months after inoculation. TIC TR: 1/185, TIC TS: 1/109. Chi-square = 0.0524. **C**) Immunofluorescence analysis for a human-specific mitochondrial (Mx) marker, the cancer marker CEA, the epithelial marker E-cadherin, and the mesenchymal marker laminin in clonal cultures derived from ovc316-XC. Clonal cultures contain human cancer cells with either epithelial (upper panel), mesenchymal (center) or both phenotypes (E/M hybrid clone, lower). **D**) Tumor-forming abilities after transplantation of 10^5^ E, M, or E/M cells into SCID-beige mice. Tumor formation was evaluated 4 months after inoculation. **E**) Xenograft tumors derived from E/M hybrid clones. The human origin of tumor cells is shown by staining for a human specific mitochondria marker (green). Staining for the epithelial antigen AE1/3 and the tumor antigen CEA reveals phenotypic heterogeneity in tumors that are derived from clonal E/M cultures. Laminin-expression is largely restricted to vascularized regions. **F**) H&E staining of a xenograft derived from an ovc316-XC E/M clone and from a patient biopsy (ovc316-biopsy) showing that xenografts from resemble the histology of human tumors *in situ*. The scale bar is 40µm.

Cultures contained different cell types, with clearly distinguishable subsets expressing different cancer markers (e.g. CA-125 or CEA), which was reminiscent of the heterogeneity of tumor cells seen from analysis of sections of the original tumor or the tumor xenograft (Figure 1A, left three panels). Notably, tumor cell heterogeneity was less pronounced in established ovarian cancer cell lines such as SKOV3-ip1 (Figure 1A, right panel).

Considering the phenotypic heterogeneity of ovarian cancer, the goal of our study was to isolate different cell subsets of primary ovarian cancer cultures and test them for parameters that have been associated with cancer stem cells, including proliferation, pluripotency, and tumorigenicity. Light microscopy analysis of xenograft cultures (e.g. ovc316-XC) revealed characteristic epithelial subsets that contained polymorphic cells, which also expressed large amounts of the extracellular matrix protein laminin at the interphase to surrounding non-epithelial cells. In culture the epithelial cellular subsets showed higher resistance to trypsin treatment (Figure 1B). We separated the cells from early passages (p<6) into two fractions based on trypsin resistance, and tested their tumorigenicity in SCID-beige mice, but no difference in tumor forming ability was observed (Figure 1B, right panel).

As done previously in an earlier study that analyzed the cellular resistance to oncolytic adenoviruses [33], we established 100 clonal cell cultures derived from early passage ovc316-XC cells. Clones were expanded and analyzed for human-specific and cancer markers (to exclude the presence of mouse stromal cells) as well as epithelial and mesenchymal markers (Figure 1C). A total of 20% of the resulting cultures were restricted to an epithelial phenotype (E-cadherin positive; “epithelial clone”), whereas 19% were mesenchymal (laminin positive, “mesenchymal clone”). The remaining 61% of clonal cultures contained both epithelial and mesenchymal cells, in addition to cells that were positive for both epithelial and mesenchymal markers (Figure 1C). We called these cells epithelial/mesenchymal (E/M) hybrid cells. Clonal epithelial and mesenchymal cultures had limited long-term proliferative potential (20–25 passages mesenchymal, 20–40 passages epithelial cultures respectively). Notably, during passaging, cells in one of the 20 epithelial clones lost membrane E-cadherin and claudin 7 and acquired the mesenchymal transcription variant of p120 catenin [33], indicating that it underwent an EMT (Figure S1).

Importantly, only cultures containing E/M cells were able to form tumors in SCID-beige mice within 4 months when 10^5^ cells (passage 6) were injected (Figure 1D). Tumors displayed phenotypic heterogeneity, contained tumor stroma and were vascularized (Figure 1E). Overall, histology of tumors derived from transplanted clonal E/M cells was similar to that of the patient tumor (Figure 1F). Serial transplantation of cells derived from these tumors also gave rise to new tumors (data not shown). Significantly, this study shows that a single cell can form a clone that is subsequently able to form a heterogeneous tumor. This indicates that the original cell was a potential cancer stem cell, with the ability to differentiate, self-renew, and form a tumor. Taken together, these data show that primary ovarian cancer cultures are phenotypically heterogenous and that tumor-initiating cells appear to have traits of mesenchymal and epithelial (E/M) cells.

### Cells at the periphery of trypsin-resistant subsets contain epithelial-mesenchymal hybrid (E/M) cells that co-stain with stem cell markers

**Figure 2:**
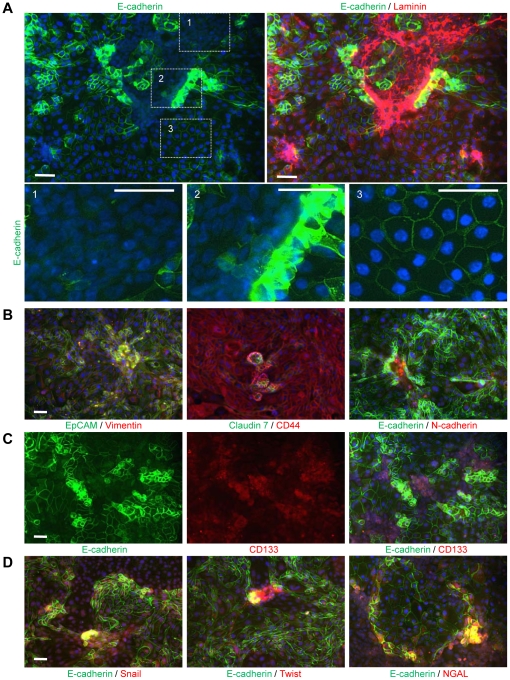
Tumorigenic primary ovarian cancer cultures contain epithelial-mesenchymal hybrid (E/M) cells that co-stain with stem cell markers. **A**) Analysis of E-cadherin (green) and Laminin (red) in ovarian cancer cultures. Magnification of marked areas is shown at the bottom: A1) E-cadherin^negative^ cells express laminin. A2) Epithelial/mesenchymal (E/M) hybrid cells: E-cadherin^low^ cells show mainly cytoplasmic localization of E-cadherin and high expression of laminin. E-cadherin^high^ cells contain elevated membrane E-cadherin levels and lower amounts of laminin. A3) E-cadherin^positive^ cells have an increased cell size and maintain lower levels of clearly membrane-localized E-cadherin than E-cadherin^high^ cells. Laminin expression is almost absent in E-cadherin^positive^ cells (upper right panel). **B**) Analysis for additional epithelial (green) and mesenchymal (red) markers confirms the existence of E/M hybrid cells in ovc316-XC cultures. Epithelial marker^high^ cells are in close proximity to mesenchymal marker^high^ cells and stain double positive in areas that contain spheres. In the left and middle panel, note that all cells are positive for vimentin and CD44. **C**) CD133 marks areas that contain E-cadherin^high^ and E-cadherin^low^ cells. **D**) E-cadherin^high^ and E-cadherin^low^ cells show strong co-labeling with the EMT-inducers Snail, Twist, and NGAL. The scale bar is 40µm. Shown are images from ovc316-XC. Immunofluorescence analysis of ovc0117-PC, ovc0122-PC, ovc100506-XC, and ovc100728-XC revealed similar results.

Having found a potential link between tumorigenic cancer stem cell-like and epithelial/mesenchymal traits on clonal cultures from ovc316-XC, we performed immunofluorescence analyses of the primary ovarian cancer cultures (derived from biopsies or xenografts) with a number of epithelial and mesenchymal markers (Figure 2). We focused our analysis on cells that were isolated from xenografts and had just (passage 0/1) or recently (passages 6–10) adapted to tissue culture, and contained large proportions of trypsin-resistant subsets. These subsets stained positive for epithelial markers E-cadherin, claudin7 and EpCAM on lateral cell membranes (Figures 2A, B). Cells surrounding epithelial subsets were negative for these markers on their lateral membranes and stained for mesenchymal proteins vimentin and laminin. Further analysis for E-cadherin revealed phenotypic diversity within ovc316-XC cultures. Two types of cells that localized E-cadherin to their membranes were observed, membrane E-cadherin^high^ and membrane E-cadherin^low^ (Figure 2A, lower panel, insert #2 and 3#). A third type of cells adjacent to membrane E-cadherin^high^ cells contained clearly detectable E-cadherin in the cytoplasm/nucleus (Figure 2A, lower panel, insert #2). Cytoplasmic E-cadherin^high^ cells also stained positive for mesenchymal cell markers such as laminin as well as caldesmon-1 (a myeloid marker), the endothelial/endothelial progenitor markers CD31, and VCAM-1 (Figure S2A). E-cadherin^high^ E/M-cells also expressed high levels of the angiopoietin receptor Tie2 (Figure S2A, right panels). Notably, Tie2 is a marker for hematopoietic stem cells that is also found on a series of epithelial tumors [34], [35].

Cultures at passage 0/1 generally showed higher overall expression of epithelial and mesenchymal markers than cultures in passages 6–10 and formed 3 dimensional cell conglomerates/spheres containing E/M hybrid cells in highly proliferative areas (Figure 2B). The E/M hybrid stage of cells (within spheres) was confirmed by double staining with other epithelial markers (EpCAM, claudin7) and mesenchymal markers (N-cadherin, vimentin). Furthermore, spheres stained for EpCAM and the mesenchymal stem cell marker CD44. Notably, most cells stained for EpCAM, CD44, and vimentin (Figure 2B), suggesting that EpCAM/CD44 signals underestimate the phenotypic diversity within early passage ovarian cancer cells. The best discrimination between different subsets was achieved with E-cadherin in combination with a mesenchymal marker or CD44. Subsequently we used this marker combination to characterize most of the ensuing experiments. When analyzed for E-cadherin and the putative cancer stem marker CD133, two subfractions of CD133 positive cells become apparent CD133^+^/E-cadherin^high^ and CD133^+^/E-cadherin^low^ cells (Figure 2C). This observation further supports the role of E-cadherin as a potential discriminatory marker for cancer cell subsets. Cells within the E/M cell population (particularly E-cadherin^low^ cells) also expressed other markers that are characteristic of stem cells, including Nanog, Oct 4, and Sox2 (Figure S2B). E/M cells, particularly cells in spheres, were also highly positive for the EMT inducers Snail, Twist and NGAL (Figure 2D). Furthermore, we also found that cells that were low in membrane-claudin 7 predominantly localize beta-catenin to the cytoplasm/nucleus (Figure S2C). Transition of beta-catenin from the cell membrane to the nucleus is an early event in EMT.

Overall, immunofluorescence studies indicate the existence of two CD133^+^ fractions; i) membrane E-cadherin^high^ and ii) cytoplasmic E-cadherin^high^/membrane E-cadherin^low^ cells. Cytoplasmic E-cadherin^high^/membrane E-cadherin^low^ cells carry traits of other cell lineages and therefore, most likely, represent primitive progenitor cells or stem cells.

### Change of phenotype, EMT and differentiation *in vitro*: flow cytometry studies

**Figure 3:**
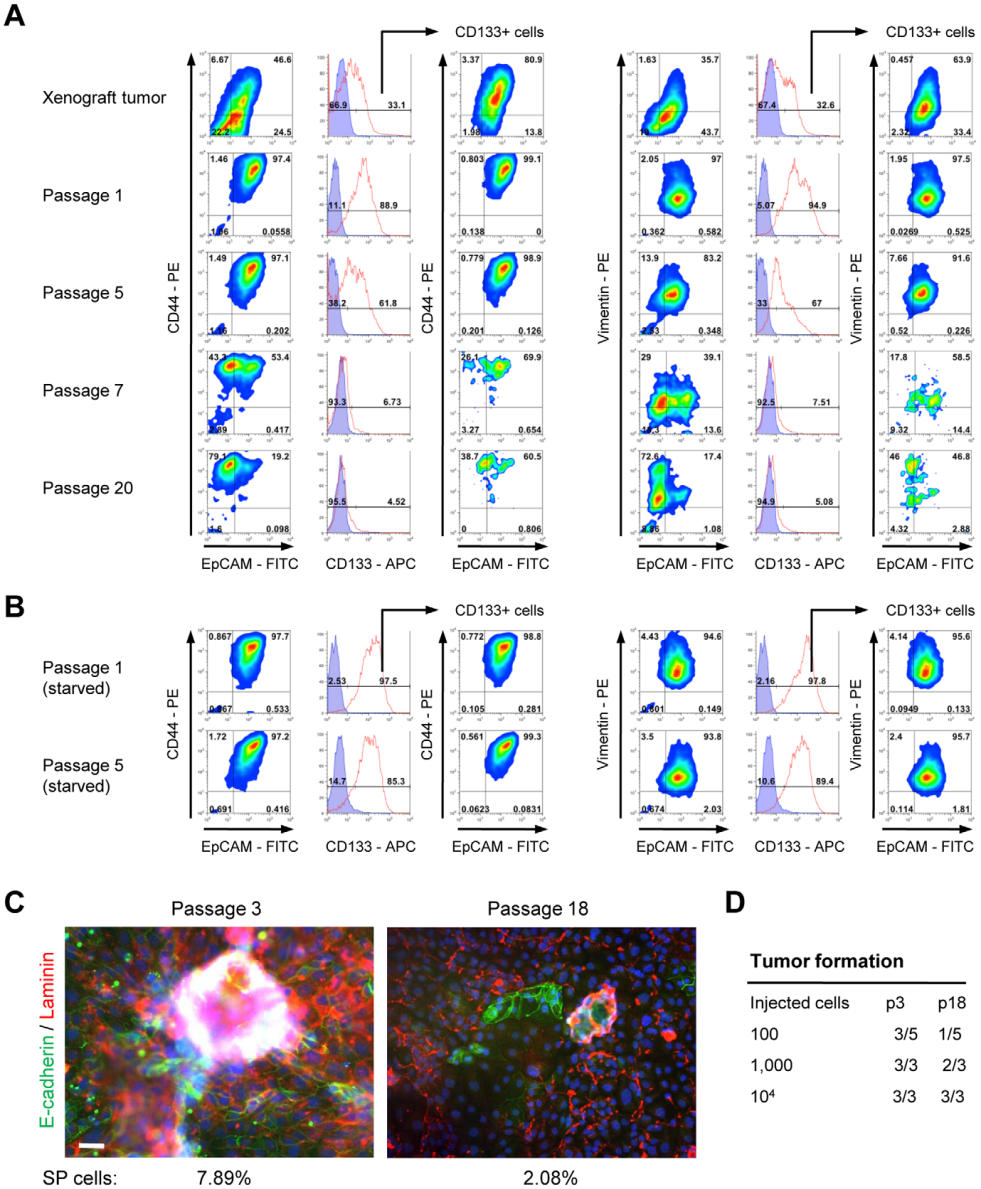
E/M hybrid cells differentiate into mesenchymal cells *in vitro*. **A**) Triple-color flow cytometry analysis of xenograft/biopsy cell suspensions and cultured cells *in vitro* at different passages. Left panel: EpCAM/CD44/CD133. Right panel: EpCAM/vimentin/CD133. **B**) Passage 1 and 5 cells were subjected to 14 days of growth factor/FCS starvation and then analyzed by flow cytometry. Shown are data from ovc316-X and ovc316-XC. The findings were confirmed on cell suspensions from biopsies and primary cultures (ovc100506-biopsy, ovc100506-PC, ovc100728-biopsy, and 100914). **C**) Immunofluorescence analysis of passage 3 and 18 cultures of ovc316-XC. Cells in early passages (passage 3) have elevated levels of E-cadherin and laminin when compared to passage 18. Cells sizes are increased and sphere-growth in high cell densities is markedly reduced in high passages. Percentage of side population (SP) positive cells are shown below. The scale bar is 40µm. **D**) Tumor formation after transplantation of ovc316-XC passage 3 and 18 cells into SCID-beige mice (evaluated 4 months after inoculation). TIC p3: 1/110, TIC p18: 1/744. Chi-square: 0.245.

To quantify numbers of cells with epithelial and mesenchymal phenotypes in cultured cells, we employed flow cytometry and started by monitoring cells for the epithelial marker EpCAM as well as monitoring for vimentin or CD44 marking cells with mesenchymal attributes. Furthermore, to delineate phenotypic changes overtime such as initiation of EMT, we analyzed different passages of ovc316-XC cells (Figure 3). These studies revealed a number of interesting observations. i) In tumor cell suspensions that were freshly isolated from xenografts, the majority of cells were either E/M cells (EpCAM^high^/Vimentin^high^, EpCAM^high^/CD44^high^) or E-cells (EpCAM^high^/Vimentin^low^, EpCAM^high^/CD44^low^), however, at passage 1 only E/M cells could be detected, indicating that the majority of cells obtained from xenografts that adapt to tissue culture are E/M (Figure 3A). This implies that, even at early passage, cultures do not adequately reflect the cellular composition of the tumor *in situ*. Passage 1 contained EpCAM^high^/CD44^high^/Vimentin^high^ and EpCAM^high^/CD44^low^/Vimentin^low^ cells. ii) Importantly during culture of cells in MEGM containing growth factors/FBS (see passages 1, 5, 7, 20), both the EpCAM^high^/CD44^high^/Vimentin^high^ or EpCAM^high^/CD44^low^/Vimentin^low^ cell types disappeared and were replaced by EpCAM^low^/CD44^high^/Vimentin^high^ cells. This demonstrates that E/M cells differentiate into mesenchymal cells *in vitro* in an EMT-like manner. iii) The majority of CD133^+^ cells are E/M hybrid cells. After passaging in culture ovc316-XC cells rapidly lose CD133 expression concomitantly with the loss of epithelial features.

We also found that for a number of passages starvation can stall differentiation and the loss of the epithelial phenotype, while levels of CD133 slightly decrease during this process (Figure 3B). Overall, we found that primary ovarian cancer cultures show more sphere formation when cultured without growth factors (Figure S3).

Having identified E-cadherin as a marker capable of differentiating cellular subsets, we repeated flow cytometry analyses with E-cadherin (Figure S3). Differentiation was also reflected by a decrease in levels of E-cadherin and laminin after passaging. Whereas low passage cultures contained high numbers of spheres, E-cadherin^high^ cells, and excessive amounts of laminin, high passage cells showed markedly reduced sphere growth and low levels of these proteins while cell size increased (Figure 3C). This was also confirmed in flow cytometry studies of passages 1, 3, and 7 (Figures S3A–C). These studies show that the mesenchymal (E-cadherin^negative/low^) sub-fraction increases during passaging and concomitant EMT. CD133^+^ cells were highly positive for CD44 (Figure S3A). We also analyzed membrane Tie2 in correlation to E-cadherin (Figure S3B). By immunofluorescence analysis, Tie2 cells have an epithelial phenotype and are lower in CD44 than CD133^+^ cells. Notably, a correlation between E/M phenotype, CD133/CD44-positivity, and tumorigenicity is also supported by flow cytometry analysis of clonal cultures (Figure S3C). Only E/M clones contained considerable amounts of CD133^+^ cells with elevated expression of CD44 (and as shown earlier, only E/M clones formed tumors). The loss of “stem cell” features during passaging is also reflected by reduction of the fraction of side population (SP) cells from 7.8±0.5% at passage 3) to 2.1±0.3% at passage 18 (Figure 3C). The SP cell phenotype has been associated with cancer stem cells in previous studies of ovarian cancer [36]. Finally the loss of CD133^+^ and E/M cells correlated with a reduced ability to form tumors (Figure 3D).

Overall, we observed a striking difference of phenotypes between cells *in vivo* and *in vitro*. Cells that adapted to tissue culture were CD133^+^ E/M cells. After passaging E/M cells differentiate into mesenchymal cells in an EMT like manner. This process is accompanied by a reduction of tumorigenicity.

### Presence of E/M cell subsets *in situ*

**Figure 4:**
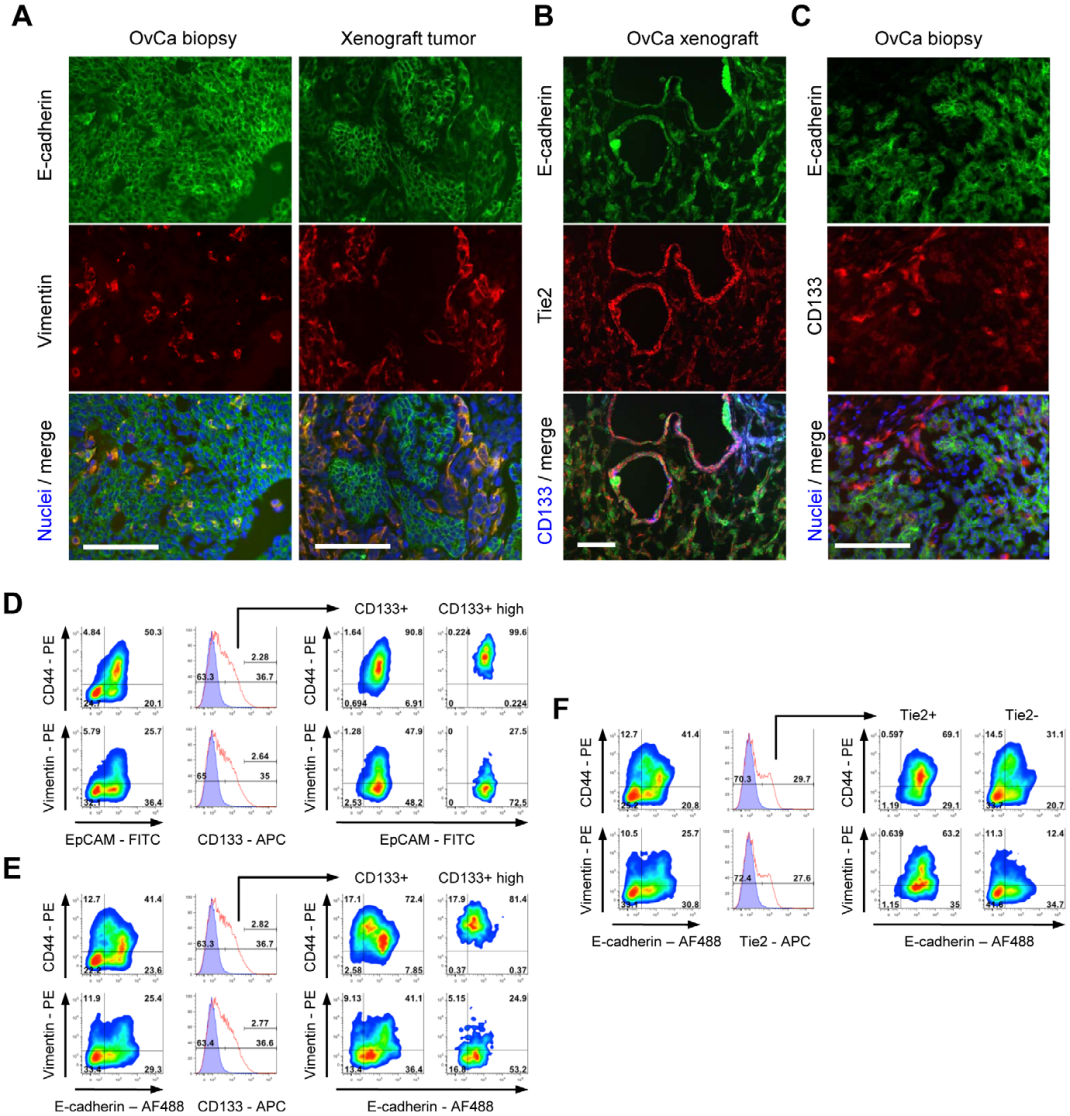
Phenotypic analysis of ovarian cancer xenografts and biopsies. **A**) Existence of E/M hybrid cells *in situ*. E-cadherin^+^/vimentin^+^ cells are present biopsies and tumor xenografts. Shown are images of ovc316-biopsy and ovc316-X. Similar staining was found for ovc100506-biopsy/ovc100506-X, ovc100728-biopsy/ovc100728-X, and ovc100914-biopsy/ovc100914-X. **B**) Correlation of E-cadherin (green) and Tie2 (red) positivity in ovarian cancer biopsies. CD133^high^ cells (blue) are Tie2^low^ and E-cadherin^intermediate^. Shown are images of ovc316-biopsy and ovc316-X. Similar staining was found for ovc100506-biopsy/ovc100506-X, ovc100728-biopsy/ovc100728-X, and ovc100914-biopsy/ovc100914-X. **C**) CD133^high^ cells have low levels of membrane E-cadherin. E-cadherin^high^ cells show punctated membrane CD133 staining. Shown are images of ovc316-biopsy and ovc316-X. Similar staining was found for ovc100506-biopsy/ovc100506-X, ovc100728-biopsy/ovc100728-X, and ovc100914-biopsy/ovc100914-X. The scale bar is 40µm. **D–F**) Triple-color flow cytometry analysis of ovc316-X for epithelial markers (x-axis), mesenchymal features (y-axis), and for cancer stem cell marker CD133 (histogram plots). **D**) Xenografts contain epithelial cells, epithelial/mesenchymal (E/M) hybrid cells, and cells that are negative for all markers. CD133^+^ cells are almost exclusively in an E/M hybrid stage based on EpCAM/CD44 analysis. CD133^high^ cells are EpCAM^high^, CD44^high^ and vimentin^low^. **E**) Replacement of EpCAM with E-cadherin reveals higher diversity within xenografts. CD44^high^ cells are E-cadherin^low/intermediate^. CD133^+^ cells are divided into two clear sub-fractions. Fraction 1: E-cadherin^low/intermediate^/CD44^high^ and fraction 2: E-cadherin^high^/CD44^intermediate^. CD133^high^ cells are E-cadherin^low/intermediate^, CD44^high^ and vimentin^low^. The positivity of CD44 and vimentin correlates inversely. **F**) Tie2 marks cells that are E-cadherin^high^/CD44^intermediate^ and predominantly positive for vimentin. Tie2^−^ cells are highly depleted for CD133 sub-fraction 2 and for E-cadherin^cells^/vimentin^high^ cells.

Taking into consideration that ovarian cancer cultures, particularly at late passages, only poorly reflect the phenotype of tumors *in situ*, we focused our immunofluorescence and flow cytometry analyses on sections of tumor biopsies or xenografts, or cell suspensions obtained by collagenase digestion of tumors (Figure 4). Immunofluorescence analyses of sections from xenografts harvested at week 10 after tumor cell transplantation (Figure 4) or sections of patient biopsies (Table S1, Figure S4) showed that tumors *in situ* have clusters of E/M cells (EpCAM^+^/vimentin^+^) as well as clusters of epithelial cells (E-cadherin^+^/Vimentin^−^) (Figure 4A). In xenografts, nests of E/M and epithelial cells were surrounded by layers of laminin. We also observed costaining of E-cadherin and Tie2 (Figure 4B). CD133^high^ cells (blue) are Tie2^low^ and E-cadherin^low^ (Figure 4B, lower panel). An interesting pattern was observed when double staining with E-cadherin and CD133 as CD133 positive cells were found at the tumor periphery, in areas of active tumor growth (Figure S5A). Notably not all CD133^+^ cells were E/M cells. Within CD133^+^ cells, we found two subsets of E/M cells as described earlier for the in vitro studies (Figure 4C, larger magnification in Figure S5B). Interestingly the CD133^high^/membrane E-cadherin^low^ subset *in situ* showed the presence of E-cadherin within cytoplasmic vesicles more apparently than *in vitro*. The results obtained using xenograft tumors were representative for staining of tumor sections from nine different patients (Table S1, Figure S4).

Immunofluorescence analyses of tumors indicate the presence of two E/M cell fractions: CD133^high^/membrane E-cadherin^low^/cytoplasmic E-cadherin^high^/Tie2^negative/low^ and CD133^intermediate^/membrane E-cadherin^high^/cytoplasmic E-cadherin^negative^/Tie2^high^.These immunohistological findings are supported by triple-color flow cytometry analysis of ovc316-X xenografts for epithelial markers (x-axis), mesenchymal features (y-axis), and for the cancer stem cell marker CD133 (histogram plots) (Figures 4D–F). Xenografts contain epithelial cells, epithelial/mesenchymal (E/M) hybrid cells, and cells that are negative for all markers. CD133^+^ cells are almost exclusively in an E/M hybrid stage based on EpCAM/CD44 analysis. CD133^high^ cells are EpCAM^high^, CD44^high^ and vimentin^low^ (Figure 4D). As in earlier studies, replacement of EpCAM with E-cadherin in analyses reveals higher diversity within xenografts (Figure 4E). CD44^high^ cells are E-cadherin^low/intermediate^. CD133^+^ cells are divided into two clear sub-fractions. Fraction 1: E-cadherin^low/intermediate^/CD44^high^ and fraction 2: E-cadherin^high^/CD44^intermediate^. CD133^high^ cells are E-cadherin^low/intermediate^, CD44^high^ and vimentin^low^. Therefore CD44 and vimentin positivity is inversely correlated. Tie2 marks cells that are E-cadherin^high^/CD44^intermediate^ and predominantly positive for vimentin (Figure 4F). Tie2^−^ cells are highly depleted for CD133 sub-fraction 2 and for E-cadherin^high^/vimentin^high^ cells.

Because the phenotype of cultured ovarian cancer cells only poorly reflects that of the tumor in situ, analysis of cell suspensions of xenograft tumors without culturing is important. These studies confirmed and extended our key conclusion from in vitro studies, namely the existence of different subsets within CD133^+^ cells: CD133^high^/membrane E-cadherin^low/intermediate^/cytoplasmic E-cadherin^high^/Tie2^negative^ and CD133^intermediate^/membrane E-cadherin^higgh^/cytoplasmic E-cadherin^low^/Tie2^positive^.

**Figure 5:**
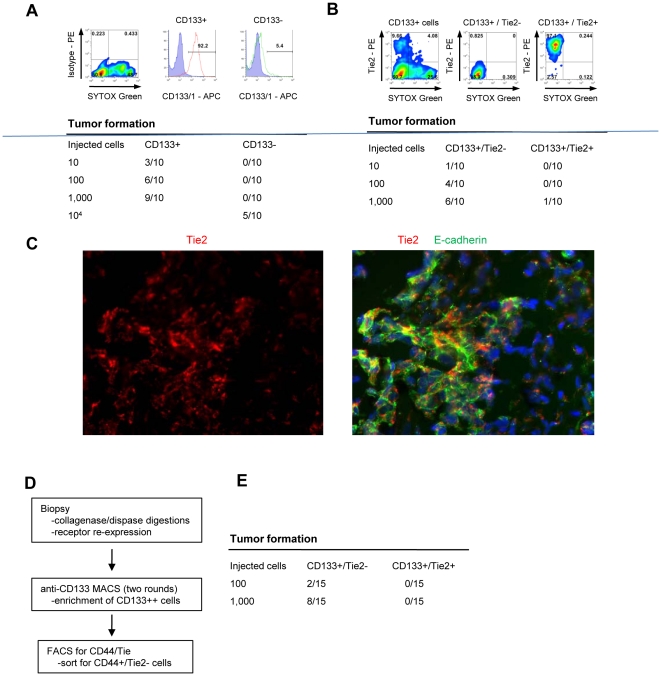
Tumor forming ability of CD133^+^ subfractions. **A**) Validation of CD133^+^ cell fractions obtained by column isolation. Purity of injected cell fractions is shown. Cells used for transplantation were sorted by FACS and non-viable cells were excluded using SYTOX Green. Tumor-forming cells are highly enriched in the CD133^+^ cell fraction. Shown are studies with CD133^+^ vs CD133^−^ cells from eight pooled ovc316-X xenografts. (N = 10 animals per group). Tumor formation was evaluated 4 months after inoculation. TIC CD133^+^: 1/191, TIC CD133^−^: 1/16725. Chi-square = 8.25, p<0.01. **B**) Column-isolated CD133^+^ cells were further subdivided into Tie2^+^ and Tie2^−^ fractions by FACS. Cells used for transplantation were sorted by FACS and non-viable cells were excluded using SYTOX Green. Tumor-forming abilities are more pronounced in CD133^+^/Tie2^−^ cells. Shown are data from transplantation of CD133^+^/Tie2^+^ vs CD133^+^/Tie^−^ cells from ovc316-XC (N = 10). TIC CD133^+^/Tie2^+^: 1/10593, TIC CD133^+^/Tie2^−^: 1/650. Chi-square: 6.74, p<0.01. **C**) Immunofluorescence analysis of tumors that developed after transplantation of CD133^+^/Tie^−^ cells. **D and E**) Analysis of fractions derived from patient biopsies. D) Schematic of cell isolation. E) Tumor formation after transplantation of CD133^+^/Tie2^−^ and CD133^+^/Tie2^+^ cells derived from ovc100506-biopsy. Tumor formation was evaluated 3 months after inoculation. TIC CD133^+^/Tie2^−^: 1185, TIC CD133^+^/Tie2^+^: n.a.

Having identified several different cellular subsets in suspensions from xenograft tumors, we attempted to isolate different fractions using MACS or FACS for subsequent tumorigenicity and pluripotency studies. Because of significant overlaps of cell populations based on epithelial (E-cadherin/EpCAM) or mesenchymal markers (CD44/vimentin), we decided to use an indirect approach to isolate E/M cell subfractions. As shown above, the vast majority of E/M cells were CD133-postive. We therefore isolated CD133-positive and CD133-negative cells using anti-CD133 conjugated magnetic bead columns (Figure 5A) and cell purity and viability was validated by flow cytometry. The “CD133^+^” fraction contained 92.2% CD133-positive cells, whereas the CD133-“negative” fraction contained 5.4% of CD133-positive cells. Tumorigenicity of CD133^+^ and CD133^−^ fractions was analyzed in SCID-beige mice. Three out of 10 mice formed tumors after transplantation with 10 CD133^+^ cells while 100 fold more CD133^−^ cells were needed to achieve tumor formation in 5 out of 10 mice (Figure 4A, lower panel). This indicates that the CD133^+^ cell fraction is enriched for tumor-forming cells. Histology of CD133^+^ and CD133^−^ derived tumors was identical, and tumors contained E/M (E-cadherin/laminin) positive cells and were vascularized (Figure S6A). This could be as a result of tumors from the “CD133^−^” population being derived from the 5.4% contaminating CD133-positive cells. Alternatively, “CD133^−^” cells might be tumor-initiating, but at a lower efficiency. Co-staining for the proliferation marker Ki-67 revealed that both CD133^+^ and CD133^−^ cells contain similar numbers of Ki-67 positive cells (Figure S6B).

Figure 4F shows that a significant subset of E/M cells were positive for Tie2 (37% of CD44^+^/E-cadherin^+^ cells or 29.3% of Vimentin^+^/E-cadherin^+^ cells) and that the vast majority of Tie2-positive cells were E/M cells (90.7% of CD44^+^/E-cadherin^+^ and 73.6% of vimentin^+^/E-cadherin^+^ cells). Instrumental for a separation of different E-cadherin fractions was the finding that the low membrane E-cadherin levels correlated with low/absent Tie2 expression. Column-isolated CD133^+^ cells were therefore further subdivided into Tie2^+^ and Tie2^−^ fractions using FACS (Figure 5B). When transplanted into mice, tumor-forming ability was clearly localized to CD133^+^/Tie2^−^ cells (Figure 5B, lower panel). Immunofluorescence analysis of tumors derived from transplanted CD133^+^/Tie2^−^ cells demonstrated that ∼50% of tumor cells were positive for Tie2 and expressed membrane E-cadherin. Tie2 negative cells had cytoplasmic, punctuated E-cadherin staining. This indicates that i) Tie2^−^/membrane E-cadherin^low^ cells transdifferentiate in vivo into epithelial Tie2^+^/E-cadherin^high^ cells (through MET) and ii) primitive Tie2^−^/cytoplasmic E-cadherin cells (i.e. potential cancer stem cells) are maintained during tumor growth.

To consolidate these findings we performed studies with tumor cells directly isolated from human ovarian cancer biopsies (see Figures 5D–F). Biopsies were digested with collagenase/dispase and cell suspensions were cultured in a rotating flask for 5 hours to restore receptor expression. Cell suspensions were then subjected to MACS for CD133 and FACS to separate CD133^+^/Tie2^−^ and CD133^+^/Tie2^+^ cells (Figure 5D). Different numbers of CD133^+^/Tie2^−^ and CD133^+^/Tie^+^ cells from biopsies were tested for tumor formation (Figure 5E). Overall, engraftment of cells from biopsies was less efficient than that of cells that had been cultured and xenografted. Importantly, however, no tumor formation was observed after transplantation of CD133^+^/Tie2^+^ cells, whereas 2 out of 15 or 8 out of 15 mice developed tumors after transplantation of 100 or 1,000 CD133^+^/Tie^−^ cells, respectively. These data indicate that tumor-forming cells are enriched in the fraction of CD133^high^/Tie2^−^/surface E-cadherin^low/intermediate^ cells.

In summary, based on the immunofluorescence and flow cytometry data *in vitro* and *in vivo*, we tentatively propose two phenotypically different subsets of CD133^+^ E/M cells: i) E/M-MP cells (CD133^high^/membrane E-cadherin^low/intermediate^/cytoplasmic E-cadherin^+^/Tie2^−^). These cells are multipotent, having epithelial, mesenchymal, and possibly other lineage traits. E/M-MP cells are enriched for tumor-forming cells. ii) E/M-E cells (CD133^intermediate^/membrane E-cadherin^high^/cytoplasmic E-cadherin^negative^/Tie2^+^). We hypothesize that these cells are epithelial progenitor cells that are less tumorigenic.

### Phenotype studies on established ovarian cancer cell lines *in vitro* and *in vivo*

**Figure 6:**
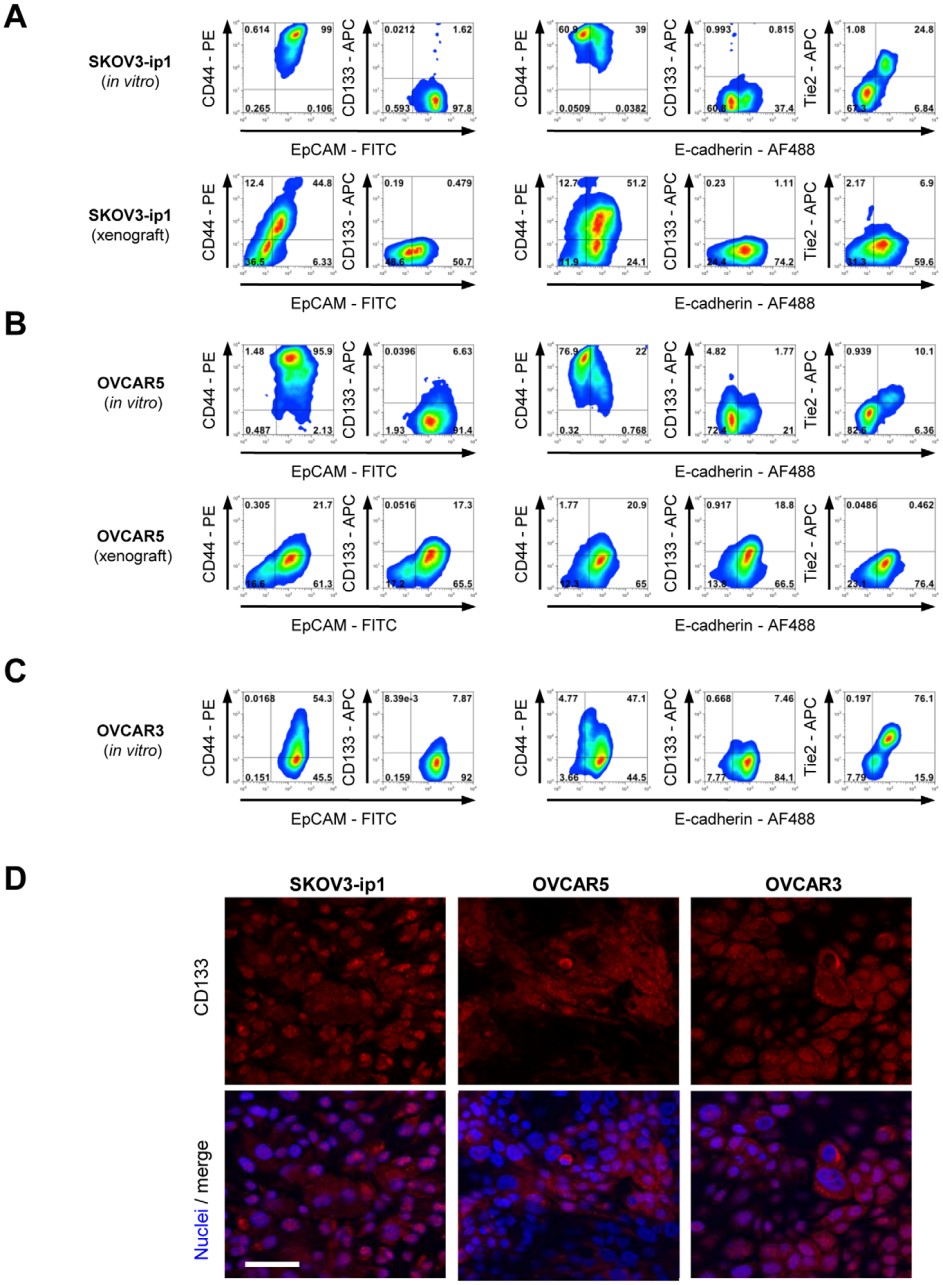
Characterization of cellular phenotypes within ovarian cancer cell lines *in vivo* and *in vitro*. **A–C**) Flow cytometry analysis for indicated markers in ovarian cancer cell lines. Upper panels show cellular phenotypes of cultures *in vitro*, lower panels show phenotypes of cells *in vivo* (xenografts). *In vitro* cultures of SKOV3-ip1 (A) and OVCAR-5 (B) contain high numbers of CD44^+^ cells and are enriched for E-cadherin negative cells. Tumor xenografts are highly epithelial. **C**) Non-tumorigenic OVCAR-3 cells have low CD44 levels and a highly epithelial phenotype *in vitro*. **D**) Immunofluorescence analysis of indicated ovarian cancer cell lines for CD133. Note that SKOV3-ip1 cells have low CD133 surface levels by flow cytometry, but contain high amounts of CD133 in the cytoplasm. The scale bar is 40µm.

We tested a series of human ovarian cancer cells lines, that are widely used as models for testing new therapeutics (SKOV-3, OVCAR3, and OVCAR5). SKOV-3 and OVCAR5 are tumorigenic while OVAR3 is not under the conditions used in this study. With regards to marker expression, SKOV-3 and OVCAR5 grouped together and both lines expressed high levels of EpCAM and CD44, but were nearly negative for surface CD133 (Figures 6A and B, upper panels). While *in vitro* the majority of SKOV3 and OVAR5 cells were E-cadherin negative, the vast majority of tumor cells in xenografts expressed high levels of E-cadherin (Figures 6A and B, lower panels). In contrast, a different marker phenotype was found for the non-tumorigenic line OVCAR3 (Figure 6C). While these cells were also high for EpCAM, CD44 levels were lower than in SKOV-3 and OVCAR5. Most OVCAR3 cells were E-cadherin-positive, having a more epithelial phenotype than SKOV3 and OVAR5 cells, which apparently inversely correlated with tumorigenicity.

In agreement with our studies in ovc316-XC cells, we found E/M hybrid cells (positive for EpCAM and/or E-cadherin, CD44^+^, and Tie2) in SKOV3 and OVC316-XC cells. We also confirmed that upon transplantation, these cells acquire a more epithelial phenotype, a process reminiscent of MET. In contrast to studies with ovc316-XC cells, the ovarian cancer cell lines were almost completely negative for surface CD133, however contained CD133 in cytoplasmic vesicles (Figure 6D), a phenomenon also recently found in studies with osteosarcoma cell lines [37]. Cells with cytoplasmic CD133 were also positive for Tie2, whereby Tie2^+^/CD133^+^ double positive cells have lower levels of CD133^+^ than CD133^+^/Tie2^−^ cells (Figure S7). In summary, established ovarian cancer cell lines displayed a hierarchical organization similar to that found in primary cultures and their derived xenografts.

### Maintenance of stem or progenitor phenotypes *in vitro* and differentiation is dependent on substrate

E/M-MP cells are enriched for tumor-forming cells and express both epithelial and mesenchymal markers (cytoplasmic E-cadherin, CD44) as well as high levels of the putative cancer stem cell marker CD133. These are all features that have been associated with cancer stem cells in the past. Other critical characteristics of stem cells in general are i) their multipotency. i.e ability to differentiate into different lineages under certain conditions, and ii) their ability to self-renew, i.e. maintain a subset of undifferentiated cells during differentiation.

#### i) EMT *in vitro* and maintenance of E/M-MP and E/M-E cells

**Figure 7:**
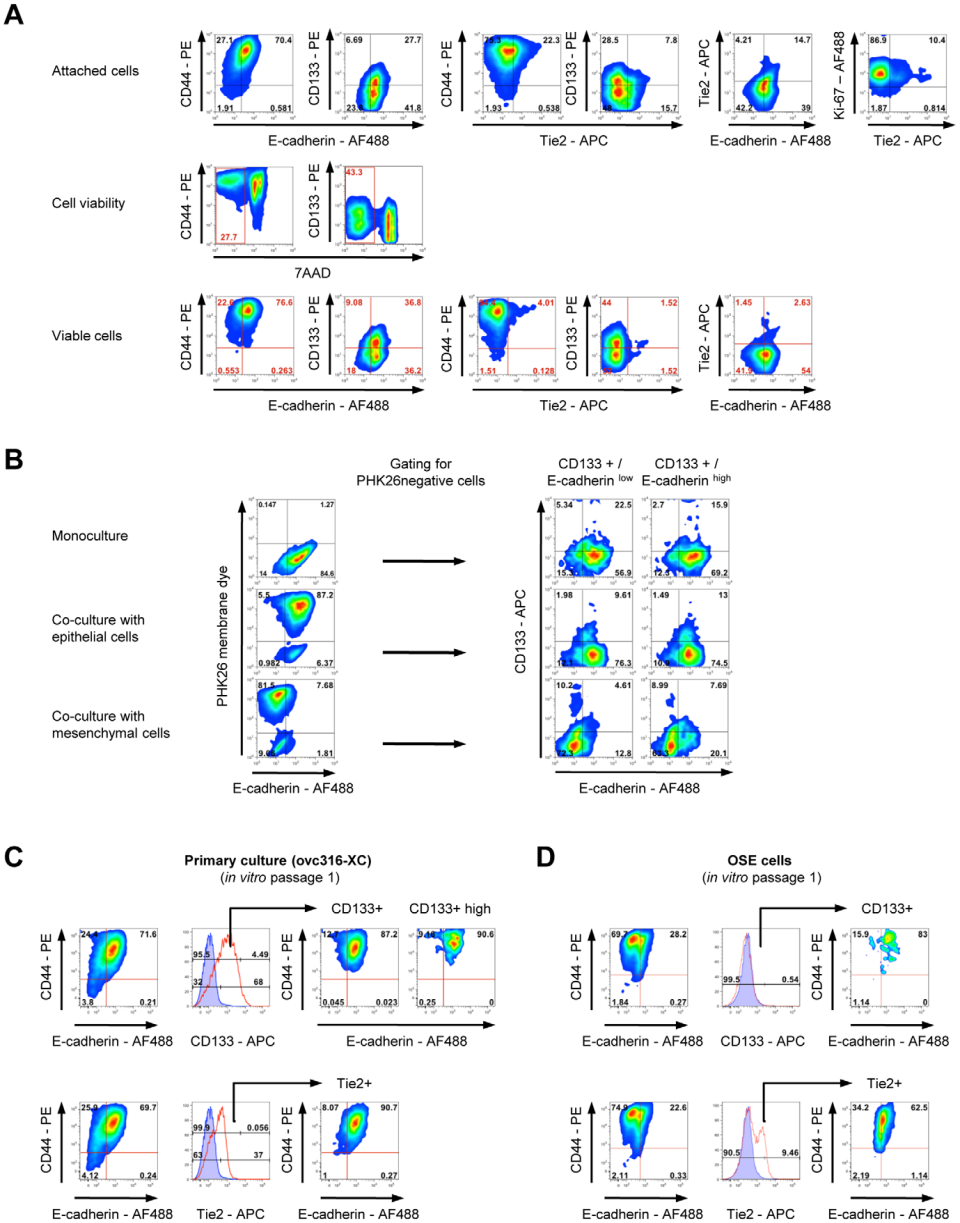
Differentiation potential of CD133^+^ cells. **A**) **Viability of different subsets.** Low passage ovc316-XC cells contain high numbers of CD133^+^ and Tie2^+^ cells. Tie2^+^/CD133^+^ double positive cells have lower levels of CD133^+^ than CD133^+^/Tie2^−^ cells. The vast majority of Tie2^+^ cells stains positive for the dead cell marker 7-AAD after treatment with versen. Viable cells show higher mean fluorescence of CD44 and CD133. All attached cells are positive for the cell proliferation marker Ki-67. **B**) CD133^+^ cells isolated from ovc316-XC tumor xenografts adapt to their environment. When cultured at low cell densities, sorted CD133^+^/E-cadherin^high^ and CD133^+^/E-cadherin^low^ cells differentiate into similar cell populations with an epithelial phenotype. When co-cultured with clonal ovc316-XC epithelial or mesenchymal cultures (ratio 1∶10), CD133^+^ cells differentiate into epithelial or mesenchymal cells, depending on the environment. Cell membrane dye PHK26 was used to discriminate between clonal cells (PHK26 positive) and cells derived from the CD133^+^ population (PHK26 negative). Cells were cultured for 7 days. **C and D**). Similarities between primary ovarian cancer cells and normal ovarian surface epithelial cells (OSE). Early passage OSE cells show high similarities with early passage ovarian cancer cultures. OSE cultures are low in E-cadherin and are CD133^+^. CD133^+^ cells within OSE and ovarian cancer cultures are enriched in cells positive for E-cadherin and CD44. Tie2^+^ cells in OSE and ovarian cancer cultures are enriched for E-cadherin and show lower mean fluorescence for CD44 than CD133^+^ cells.

Our finding that, during passaging, E/M cells give rise to cultures that predominantly contain mesenchymal cells and lost surface CD133 indicates differentiation (Figure 3). Notably, during this process, a small subset of CD133^+^ cells is maintained and this subset expresses low to high levels of surface E-cadherin and high levels of CD44. The maintenance of potential cancer stem cells also explains the development of tumors after transplantation of passage 18 cells (see Figure 3D). Interestingly, when passage 18 cultures were subjected to stress in the form of serum/growth factor starvation, E/M-cells regain features of stem cells, particularly the expression of nuclear Nanog, indicating a potential reversion of E/M-E to E/M-MP cells (Figure S8). To gather further evidence for differentiation of E/M subsets, we analyzed early passage ovc316-CX cells for cell viability after detachment using versene (Figure 7A). In agreement with earlier experiments, we found high numbers of CD133^+^ and Tie2^+^ cells, whereby the majority of CD133^+^ and Tie2^+^ cells are mutually exclusive. Tie2^+^/CD133^+^ double positive cells have lower levels of CD133^+^ than CD133^+^/Tie2^−^ cells. The vast majority of Tie2^+^ cells stain positive for the dead cell marker 7AAD after treatment with versene, indicating their loss of resistance to anoikis after detachment, and therefore a higher degree of differentiation. Viable cells show higher mean fluorescence of CD44 and CD133. All attached cells are positive for the cell proliferation marker Ki-67.

#### ii) MET *in vivo*

Another indication of differentiation comes from xenotransplantation studies. Cells used for transplantation were in passage 1 to 3 and the vast majority of cells were E/M cells. Importantly, the tumors that were derived from these cells were comprised of clusters of E/M cells (E-cadherin^+^/vimentin^+^ and EpCAM^+^/vimentin^+^) as well as clusters of epithelial cells (EpCAM^high^ or Ecadherin^high^). The presence of epithelial cells in tumors, i.e. a cell type that was absent in the inoculated cells, indicate that E/M cells differentiate towards epithelial cells *in vivo*, in a process reminiscent of MET. Maintenance and differentiation of cancer stem cells is also supported by the finding that transplantation of Tie2^−^ cells resulted in tumors consisting of Tie2-positive epithelial cancer cells and Tie2^−^ cells (representing less differentiated cells or cancer stem cells).

#### iii) Differentiation of E/M-MP cells depending on substrate (Figure 7B)

To further support differentiation of E/M cells *in vitro*, we isolated CD133^high^ cells from tumor xenografts and sorted them for viable CD133^high^/E-cadherin^low^ and CD133^high^/E-cadherin^high^ cells. When cultured at low cell densities, sorted CD133^high^/E-cadherin^high^ and CD133^high^/E-cadherin^low^ cells differentiate into similar cell populations with a mainly epithelial phenotype and low CD133 levels. We then co-cultured the two different cell fractions with either epithelial or mesenchymal cells. For the latter we used epithelial (E) and mesenchymal (M) clonal cultures (see Figure 1C). Cell membrane dye PHK26 was used to discriminate substrate E or M cells (PHK26 positive) and cells derived from the sorted CD133^+^ populations (PHK26 negative). When co-cultured with E or M cells at a ratio of 1∶10, CD133^high^ cells differentiate into epithelial or mesenchymal cells, depending on the provided environment. Notably, sorted CD133^+^/E-cadherin^high^ generated more E-cadherin-positive cells than sorted CD133^+^/E-cadherin^low^ cells when cultured among M cells (Figure 7B).

A gain in epithelial features, particularly in E-cadherin is thought to occur early in ovarian cancer development. We therefore studied the phenotype of non transformed ovarian surface epithelial cells. Importantly, a similar subset of CD133^+^/E-cadherin^+^/CD44^+^ also exists in early passage ovarian surface epithelial (OSE) cells (Figures 7C and D). This subset comprises less than 1% of the total culture. In striking difference to ovc316-XC cells, E-cadherin levels for the vast majority of OSE cells are low. Tie2^+^ cells in OSE and ovarian cancer cultures are enriched for E-cadherin and show lower mean fluorescence for CD44 than CD133^+^ cells.

### Activation of EMT pathways during passaging of ovc316-XC cells

**Figure 8:**
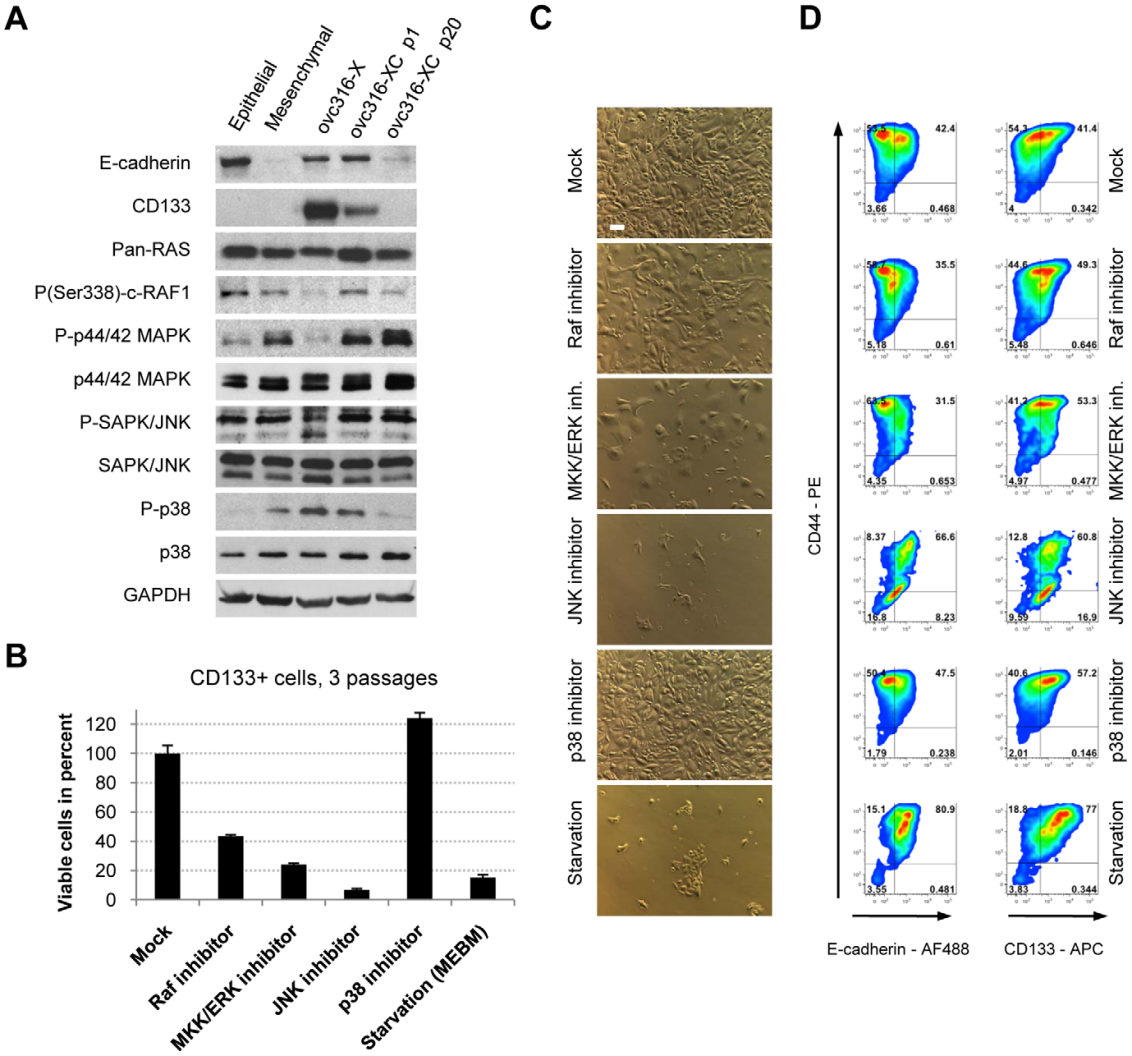
Impact of MAP kinases on proliferation and differentiation in CD133^+^ derived cells. **A**) Western blot analysis of E-cadherin, CD133 and the MAPK pathway in ovc316-XC tumor xenografts, low passage (p1), high passage (p20), and in clonal epithelial and mesenchymal cultures. Xenograft tumors contain high amounts of E-cadherin and CD133, which decrease after culturing. Concomitantly with a loss of these markers occurs a switch from phophorylated p38 to phophorylated ERK44/42. **B**) Impact of MAPK pathway inhibitors on proliferation. Cells (p7) were passaged in the presence of inhibitors for 21 days. **C**) Bright field analysis of ovc316-XC cells cultured in the presence of indicated inhibitors after 21 days. The scale bar is 40µm. **D**) Flow cytometry analysis of ovc316-XC cells cultured in the presence of indicated inhibitors after 21 days (N = 3).

The finding that E/M cells undergo EMT *in vitro* is supported by the analysis of signaling pathways using Western blot with antibodies to key kinases (Figures 8A). It is generally accepted that EMT is characterized by the activation of several kinases, including ERK1, ERK2, and PI3K/AKT [17]. Xenograft tumors contain high amounts of E-cadherin and CD133, which decrease after culturing. Concomitant with a loss of these markers is a switch from phophorylated p38 to phophorylated ERK44/42. Adaption to tissue culture (compare ovc316-X tumor and ov316-XC p1) is also associated with ERK44/42 and cRAF1 activation. The decrease in the phosphorylated form of the stress kinase p38 can most likely be explained by the loss of ECM and integrin signaling when cells are removed from tumor xenografts.

The activation of pathways during *in vitro* passaging is further corroborated by studies using specific phosphokinase inhibitors (Figures 8B–D). The inhibition of Raf, MKK, or JNK significantly decreases proliferation rates of ovc316-XC cells. The inhibition of p38 increases the number of viable cells. Flow cytometry shows the presence of the two E/M-MP and E/M-E (E-cadherin^high^/CD44^high^ or E-cadherin^low^/CD44^high^) fractions (Figure 8D). Inhibition of JNK, Raf, and growth factor starvation decreases the mean level of CD44. Growth factor starved and JNK-inhibited cells are highly epithelial and CD133 positive indicating that JNK pathways are also involved in mediating EMT. In the presence of JNK inhibitor, cells differentiate into epithelial cells/progenitors (CD44^low^, CD133^intermediate^) instead. The inhibition of Raf and MKK/ERK highly depletes ovc316-XC cultures for E-cadherin^high^/CD44^high^ cells, indicating that Raf and ERK are important for proliferation and maintenance of undifferentiated epithelial cells (E-cadherin^high^/CD44^high^). The inhibition of p38 increases the mean level of CD44, demonstrating a role for p38 in the maintenance of undifferentiated epithelial cells (E-cadherin^high^/CD44^high^). Taken together, pathway and inhibitor analyses support the presence of EMT *in vitro* upon culturing of ovarian cancer cells.

## Discussion

**Figure 9:**
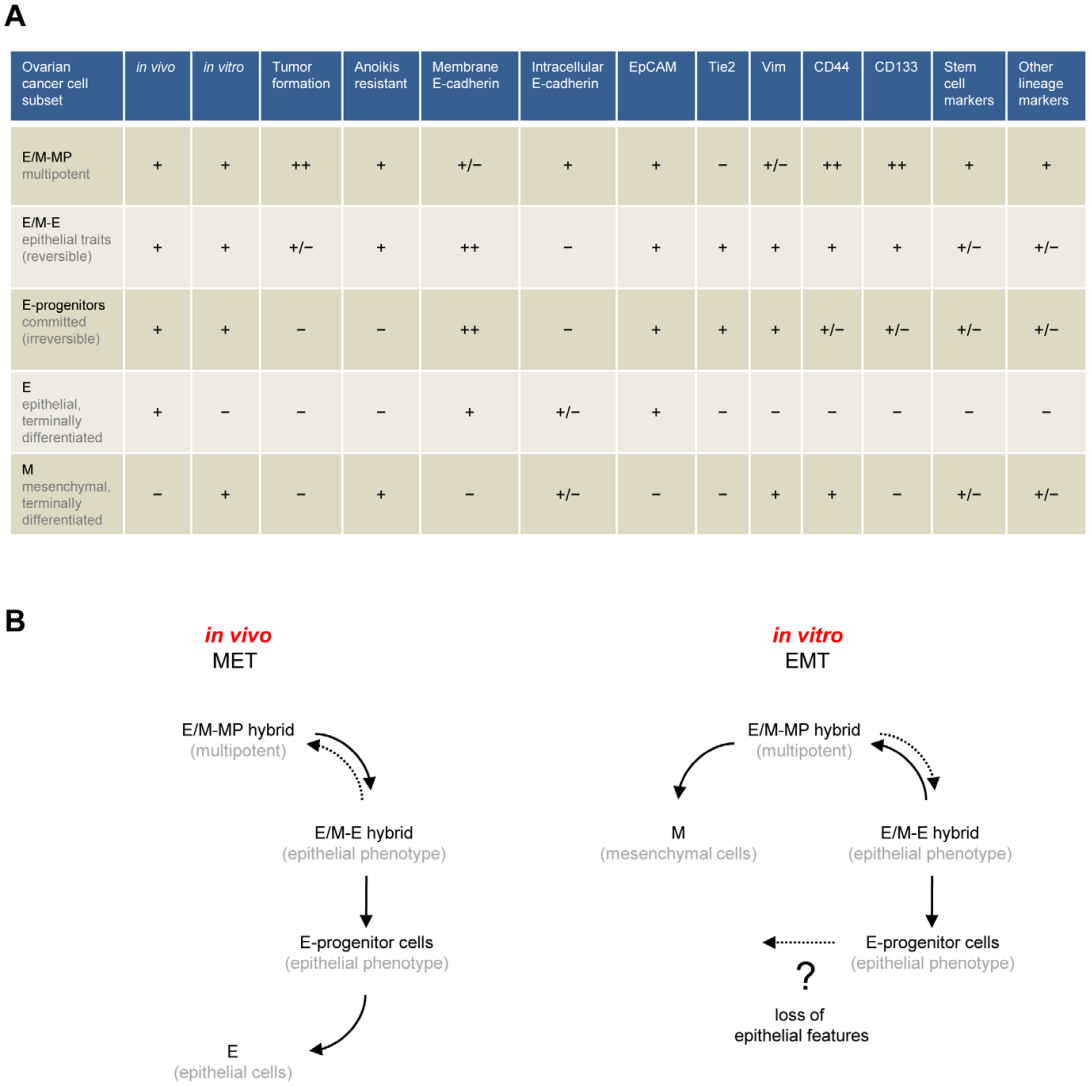
Cellular subsets in human ovarian cancer. **A**) Summary of phenotypic markers found in different ovarian cancer subsets. **B**) Interconnection between different subsets. (Description in text).

For this study we investigated the characteristics of human ovarian cancer cells derived from patient tumor biopsies. Our conclusions are based on the analysis of 14 different biopsies, 10 primary ovarian cancer cultures, 4 xenografts and cultures thereof. In the different cellular subsets found within these tumors we noticed a number of phenotypic features, which are summarized in Figure 9A. Our best understanding of the interconnections between these diverse subsets in relation to their relative mesenchymal or epithelial status is described in Figure 9B. In primary ovarian cancer cultures and tumors *in situ* we found cells that were in an epithelial and mesenchymal (E/M) hybrid stage. Based on the differences in the levels of the markers E-cadherin, CD133, Tie2 and CD44, we defined different subsets within E/M cells that differed in pluripotency and tumorigenicity. A multipotent, tumorigenic E/M cell subset (E/M-MP), that fits the profile of true cancer stem cells, had high levels of cytoplasmic E-cadherin, membrane CD133 and membrane CD44. This subset also expressed markers of other lineages, including CD31, VCAM-1 and caldesmon, and displayed features of stem cells, including the presence of nuclear-localized Nanog. MACS and FACS based separation of this subset was possible based on the presence of CD133 and CD44, and the absence of Tie2. Several lines of data indicate that these E/M-MP cells are multipotent. We found that transplantation of CD133^high^/membrane E-cadherin^low/intermediate^/cytoplasmic E-cadherin^+^/Tie2^−^ resulted in tumors consisting of membrane E-cadherin^high^/Tie2-positive cells, indicating that tumor growth *in vivo* is driven by E/M-MP cells, which give rise to an E/M subset, with more dominant epithelial features (E/M-E), and subsequently to epithelial cells that lacked all mesenchymal and stem cell traits, and that had lost the ability to form tumors (when isolated *in vitro* in the form of clonal cultures) (Figure 9B, left scheme). Therefore, tumor growth in xenografts appears to involve processes reminiscent of MET. Corresponding MET-inducing signals might emanate from the tumor-associated stroma, be due to the relative lack of growth factors (compared to *in vitro* cultures), and/or be caused by the 3-dimensional organization of tumor cells.

In contrast, we found *in vitro* indication of E/M-MP cells differentiating into mesenchymal cells, through the process of EMT (Figure 9B, right scheme). Of all the cells in suspensions derived from patient biopsies or xenografts, only E/M hybrid cells (E/M-MP and rare E/M-E) adapted to tissue culture and were found in early cell passages. After passaging these E/M cells differentiated into mesenchymal cells that lacked epithelial or stem cell markers and tumor forming ability. Our findings are suggestive for EMT, however could theoretically also be due to selective expansion of specific cell subsets. The following data support EMT rather than selective expansion: i) E/M cells express EMT inducers such Snail, Twist and NGAL (Figure 2D), ii) in vitro culturing is associated with the activation of EMT pathways (ERK1, ERK2, and PI3K/AKT) during culturing and passaging (Figure 8), iii) Studies with clonal cultures showed that epithelial cells can lose membrane E-cadherin and claudin 7 and acquire the mesenchymal transcription variant of p120 catenin (Figure S1), and iv) the same CD133^+^ E/M cells give rise to either epithelial or mesenchymal cells when cultured on epithelial or mesenchymal substrates, respectively (Figure 7B). We speculate that activation of EMT programs *in vitro* is facilitated by the disruption of cell-cell adherens junctions, due to culture medium supplements such as EGF, and the cell-ECM adhesions mediated by integrins.

Interestingly, E/M cells isolated from xenografts seem to be initially primed towards epithelial differentiation for a number of passages, as supported by several experiments (Figure 3, Figure S3A). Here, the maintenance of high CD133 levels and self-renewal of undifferentiated cells depends on high cell densities (Figure 3, Figure S3A). The accelerated cell division of undifferentiated E/M cells might be supported by the high production of extracellular matrix proteins, observed in early passages (Figure 3C), whereas low cell densities of E/M-MP cells, or growth among more differentiated cells, enforces differentiation and the rapid loss of CD133 (Figures 7A,B). Moreover, the phenotypic plasticity of E/M-MP cells and their daughter cells highly depends on the provided cellular environment. During *in vitro* passaging, EMT-inducing events trigger the generation of E-cadherin^negative^ cells, which is accompanied by the loss of CD133 and lowered secretion of ECM components (Figure 3). The culturing of E/M-MP cells among mesenchymal cells accelerates the loss of E-cadherin, whose maintenance on the cell surface requires homophilic intercellular connection.

Our data also indicates that the composition of E/M subsets fluctuates when cultures are exposed to stress such as starvation. Cultures that were subjected to starvation become enriched for cells with stem cell features (e.g. presence of Nanog in nuclei, shift from membrane E-cadherin to nuclear E-cadherin). This indicates that E/M-E cells can revert to E/M-MP cells and vice versa *in vitro* and potentially *in vivo* (dashed arrows, Figure 9B). Furthermore, our *in vitro* data suggests the existence of committed epithelial progenitor cells (membrane E-cadherin^high^, CD133^intermediate/low^, CD44^intermediate^, Tie2^+^) that lost resistance to anoikis and the ability to revert into E/M-E and E/M-MP cells. We also speculate that these cells might be able to lose E-cadherin and convert, through a secondary EMT, into more differentiated mesenchymal cells (Figure 9B, dotted arrow). A similar process is thought to occur late in ovarian cancer development and during ovarian cancer metastasis. As discussed earlier, subfractions and processes similar to those described by us for primary ovarian cancer cells appear to exist in the development of ovarian cancer.

A central conclusion from our studies of ovarian cancer is the existence of E/M hybrid cells. Our data suggest that specific E/M subsets are pluripotent, i.e. have the ability to transdifferentiate into epithelial and mesenchymal cancer cells through MET and EMT, respectively. EMT in the context of the CSC concept in breast cancer was recently reported by Weinberg's group. They found that induction of EMT through over-expression of Snail or Twist in human mammary epithelial cells caused these cells to exhibit cell surface markers similar to those of cancer stem cells, such as CD44^high^/CD24^low^ [38]. These and other studies on prostate cancer [39] indicate that EMT-like events, i.e. the generation of mesenchymal-like cells, are associated with an increase in tumor-forming capacity. In contrast, we found in our studies with ovarian cancer cells that cultures, which predominantly contain mesenchymal cells are less tumorigenic than E/M cultures from which they are derived from. We speculate that this discrepancy is due to intrinsic differences between ovarian cancer and other epithelial cancers [25].

We also found that E/M-MP cells have endothelial traits (CD31, VCAM-1). Notably, two recent publications have shown that glioma stem cells can differentiate into endothelial cells and generate tumor vasculature [40], [41].

Furthermore, our data suggest that ovarian cancer displays both a spatial and temporal heterogeneity involving tumor cell subsets that differ in their ability to form tumors and differentiate. Ovarian cancers are composed of tumor-driving E/M-MP cells as well as non-tumorigenic differentiated cancer cells that compose the bulk of cells in a tumor. This observation has implications for tumor therapy, which is often directed toward markers found on epithelial cancer cells (e.g. Her2/neu, EGFR, FGFR).

Future studies will have to focus on the delineation of the genetic and epigenetic differences between cellular subsets. These studies will also clarify the currently widely discussed question about whether tumor heterogeneity is due to distinct clones from different CSCs or whether CSCs, like their normal counterparts, are multipotential. A conclusion from our studies is that the heterogeneity of malignant cells present in ovarian cancer is the result of the multipotential capacity of E/M-MP cells for differentiation.

The concept of a hierarchy of pluripotent progenitors that fluctuates during tumor growth and under selective pressure is not without precedents. Studies on leukemia-initiating cells indicate that there is probably not a single cancer stem cell, but different groups of stem cells that can change their phenotype depending on external factors. Studies in xenograft models have shown that multiple subclones capable of leukemia initiation arise in parallel, and that most of the clones that initially form a tumor do not survive long term and do not have the capacity for self-renewal because they disappeared upon retransplantation [3].

Until recently, it was thought that tumor-initiating cells are a rare subpopulation in cancers. Nevertheless, improvements in xenotransplantation models and the use of syngeneic mouse models of breast cancer have shown that the number of tumor-initiating cells is much larger [16]. It is still elusive whether these findings can be generalized to other cancers, including ovarian cancer. Our findings that large percentages of E/M cells have tumor-forming abilities support this. Notably, the CSC hypothesis does not rely on the size of the cancer-initiating population, but more importantly on the presence of a hierarchy of tumor-forming, multipotent cells.

CD133 has been used to enrich for cells with tumor-initiating ability from a variety of human solid tumors from the brain, prostate, liver, breast and colon [42], but there are also studies that question the value of CD133 as a putative CSC marker, particularly in colon cancer and melanoma [16]. Few attempts have been undertaken to further characterize the CD133^+^ population of cancer cells. In human breast cancer cell lines, it has been found that an ALDEFLUOR-positive CD44^+^ subpopulation of CD133^+^ cells was more enriched for tumor-initiating cells than ALDEFLUOR-negative CD44^−^/CD133^+^ cells [42]. In osteosarcoma, a CD133^+^/ABCG2^+^/SP^+^ subset was enriched for cells with CSC features, such as the formation of spheres/clusters in serum-free medium with high clonogenic efficiency [37]. A clonal cell expansion model in glioblastoma recently revealed multiple tumor-initiation populations with different potential for hierarchical differentiation and aggressiveness. These populations were either positive or negative for CD133, whereby one fraction of CD133 negative cells could also give rise to CD133^+^ cells [43]. Our study confirms earlier reports that in ovarian cancer the CD133^+^ population is enriched in tumor-forming cells when compared to CD133^−^ cells [11], [44]. We also found that in biopsies from different ovarian cancer patients (Figure S4) and xenograft tumors (Figure 3), CD133^+^ cells comprise a large fraction (∼30%) of tumor cells. This determines that further differentiation of cells within the population of CD133^+^ cells is needed. Our study is the first to utilize E-cadherin and Tie2 as a marker to isolate different CD133^+^ fractions from tumor material. Potentially, other endothelial markers (e.g. VCAM-1) could be used for a similar purpose. Notably, even though CD133^+^/Tie2^+^ fail to efficiently initiate tumor growth in mice, it remains questionable whether these cells also have a growth disadvantage in established tumors. The positivity for proliferation marker Ki-67 among CD133^+^ cells in xenografts would argue against this (Figure S6B). Furthermore, it is possible that tumors arise at a later time point, since primary ovarian cancer cells from patient biopsies display much greater heterogeneity than established cancer cell lines. Ultimately, the analysis of tumor-forming abilities most likely resembles only a “snapshot” of tumors or tumor cell cultures at time of analysis. Such analyses might therefore underestimate the potential of less tumorigenic cells that have the potential for de-differentiation under different conditions.

In our study the loss of CD133 was linked to EMT in *in vitro* cultures. Surface CD133^+^ mesenchymal cells were rarely observed, whereas mesenchymal cells often harbored CD133 in the cytoplasm. Notably, CD133 has been recognized as a marker for developing epithelium [45]. Therefore, the isolation of cells from (mainly mesenchymal) *in vitro* cultures based on surface CD133 might not assess the complete tumor-forming population. An important conclusion from our study is that CD133 mainly marks a population of E/M hybrid cells, which is adaptable to different conditions and able to differentiate into either epithelial or mesenchymal cells depending on the provided environment. We therefore suggest that CD133 should only be used in combination with epithelial and mesenchymal markers in order to identify and study tumor-initiating cells within ovarian cancers.

The overall picture of potential cancer stem cells that we obtained from our study is relatively complex. It involves the existence of subsets of tumor forming cells with distinct differentiation potential. The composition of these subsets is transitory, and can shift bi-directionally, depending on external conditions and the stage of tumor growth. The understanding of this phenotypic complexity has important implications for ovarian cancer therapy.

## Methods

### Tissue Culture

Ovarian cancer biopsies were provided by the Pacific Ovarian Cancer Research Consortium (POCRC) Specimen Repository without any cconfidential information which would serve to identify a patient (Fred Hutchinson Cancer Research Center IRB protocol # 6289). Tumor tissue from biopsies was dissected into 4mm pieces and digested for 2 hours at 37°C with 1mg/ml collagenase/dispase (Roche) and 0.1mM CaCl_2_ (Sigma) in RPMI 1640 (Gibco). This was followed by incubation with versene (Gibco) (1∶1 vol/vol) for 1 hour. The protease digestion was stopped by the addition of FBS (Gibco) to a final concentration of 10%. Cells were passed through a 70 µm cell strainer (BD Falcon) using the plunger of a 5ml syringe (Becton Dickinson). The cell strainer was washed with 25ml RPMI and the total flow-through was pelleted. The pellet was resuspended in 5ml RPMI/10%FBS with 1mg/ml DNaseI (Roche) and incubated for 30 min at 37°C. Cells were pelleted again and subjected to a ficoll gradient (10ml underlay, Stem Cell Technologies) separation step if high amounts of erythrocytes were observed. The resulting cultures were kept in MEGM (Lonza) at low serum levels (1–2%) and in high cell densities, in order to minimize fibroblast growth. Clonal ovc316-XC cultures were established as published previously [33]. Clones were established by limited dilution (0.5 cells seeded per well in a 96 well plate) to ensure single cell suspension. Single cell plating was confirmed by light microscopy and wells containing more than one cell were excluded from further culturing. Further rounds of subcloning of clonal E/M cultures yielded the same secondary and tertiary clonal cultures with hybrid E/M phenotype and tumor-forming ability. SKOV3-ip1, OVCAR-3, and OVCAR-5 cells were cultured in MEGM/DMEM (Lonza/Gibco, 50%/50% v/v) supplemented with 10% FBS and 1% Penicillin/Streptomycin.

### Inhibitors

CD133^+^ cells were isolated by column isolation (Miltenyi Biotec). A total of 2.5×10^5^ CD133^+^ cells were seeded in 12 well plates (BD Falcon) and subsequently treated with inhibitors diluted in fresh MEGM medium. All inhibitors were purchased from Calbiochem and used in the following concentrations: Raf1 inhibitor (20 µM), U0126 (40 µM), p38 inhibitor III (10 µM), JNK inhibitor II (100 nM). Inhibitors were replaced with fresh medium every 48 hrs and cells were passaged (1∶2), when the first experimental culture (p38 inhibitor-treated) reached 100% confluence. Cells were analyzed by flow cytometry after 21 days. In order to evaluate proliferation rates, 10% (v/v) of each individual experimental setup from passage 3 were seeded into 96 well plates in triplicate. Cells were further cultured in the presence of inhibitors for 3 days prior to readout by MTT assay.

### MTT assay

MTT assays were carried out in 96 well plates in triplicate. A total of 15µl of a stock solution of 5mg/ml 3-(4,5-Dimethylthiazol-2-yl)-2,5-diphenyltetrazolium bromide (Sigma) per well was applied for 2 hrs. Medium was aspirated, formazide crystals were disolved in DMSO, and absorbance was measured at 495 nm using a plate reader. The percentage of viable cells is relative to mock treated cells.

### Flow cytometry

Adherent cells were detached from tissue culture plates by treatment with Versene (Gibco) for 30–60 min. Cells were then transferred into 15ml tubes (BD Falcon) and washed with RPMI 1640 supplemented with 10% FBS. Cell pellets were resuspended in ice-cold PBS with 1% FBS in order to block unspecific antibody binding. Then 2×10^5^ cells were incubated with antibodies in 5ml round bottom tubes (BD Falcon) in a total of 100µl for 45 min on ice. All subsequent incubation steps were carried out in the dark. Cells were washed with 3 ml PBS+1%FBS and centrifuged at 400g for 5 min at 4°C. After surface antigen staining, cells were fixed with 4% paraformaldehyde for 15 min on ice. Following a PBS+1%FBS wash, cells were either subjected to flow cytometry analysis or prepared for intracellular antigen staining (vimentin, Ki-67) by treatment with 0.1% Triton×100 (Sigma) for 15 min at room temperature. If needed, fluorophor-labeled secondary antibodies were used against the appropriate host. Samples were filtered (70µm, Becton Dickinson) and then analyzed using either a BD FACSCanto or BD FACScalibur flow cytometer (Becton Dickenson). Unspecific background of individual channels was determined with isotype controls and color compensation was done on single color-stained samples. Dead cells were excluded with 7AAD (Invitrogen). Figures were generated using FlowJo for Macintosh (Tree Star, Inc.).

### Animal studies

All experiments involving animals were conducted in accordance with the institutional guidelines set forth by the University of Washington and the Federation of European Laboratory Animal Science Associations for the care and use of laboratory animals. The studies were approved by the University of Washington Institutional Animal Care and Use Committee (protocol # 3108-01) and the Danish Cancer Society (protocol #2009-19). All mice were housed in specific-pathogen-free facilities. To establish subcutaneous tumors, CD17-SCID-beige mice were injected in the mammary fat pad with 1×10^5^ tumor cells or indicated cell numbers using a Matrigel (BD Biosciences)/MEGM (50µl/50µl) mix. Immunofluorescence analyses of tumor sections was performed as described elsewhere [46].

Limiting dilution analyses were carried out using Extreme Limiting Dilution Analysis (ELDA) [47]. Estimated numbers for frequency of tumor-initiating cells (TIC) were calculated by confidence intervals. Tumor-initiating cell populations were compared by likelihood ratio tests and obtained chi-square and p-values are indicated.

### Cell sorting

Cell sorting was performed on a pool of 8 freshly dissected ocv316-X tumor xenografts. CD133^+^ and CD133^−^ cells were isolated using the CD133/2-biotin antibody and anti-biotin microbeads according to the manufacturers protocol (Miltenyi Biotec). Three separate MACS LS columns were consecutively used to isolate high purity fractions. Following isolation, cells were either incubated with Tie2-PE antibodies or isotope control. Cell separation and cell number sorting was done with a BD Cytopia Influx. Dead cells were excluded with SYTOX Green (Invitrogen). Cells were sorted into ice-chilled 96well round-bottom plates with a Matrigel/MEGM (50µl/50µl) mix and subsequently injected into mice. Purity of sorted fractions was confirmed using the BD FACScalibur flow cytometer. For separation of CD133^high^/E-cadherin fractions the CD133/1-PE and E-cadherin-APC antibodies were used with the Beckman-Coulter MoFlo XDP. Viable cells were isolated by sorting for 7AAD negative events. Additional studies were performed with tumor cell suspensions isolated from patient biopsies. Tumors were microdisected, homogenized and digested with collagenase/dispase as elsewhere http://www.miltenyibiotec.com/download/protocols_gentlemacs_en/1624/gentleMACS-tumor_human-01.pdf. To re-express cell surface molecules, tumor cells were cultured in suspensions in a rotating tube for 5 hours and then subjected to MACS and FACs as described above.

### Co-culture of CD133^high^ cells with epithelial or mesenchymal cells

1×10^4^ sorted CD133^high^/E-cadherin^high^ or CD133^high^/E-cadherin^low^ cells were cultured either alone or in co-culture with 1×10^5^ epithelial or mesenchymal cells derived from clonal ovc316-XC cells in 6 cm dishes (Nunc). Clonal ovc316-XC cultures were labeled using the PHK26 membrane stain (Sigma) prior to co-culturing. Cell-labeling was carried out according to the manufacturers protocol with an extended labeling time of 15 min followed by three wash steps.

### Western Blot

Xenograft tumor tissue was dissected, manually homogenized (glass homogenizer) and incubated for 30 min in protein lysis buffer (20mM Hepes (pH 7.5), 2mM EGTA, 10% glycerol, 1% Triton×100, 1mM PMSF, 200µM Na_3_VO_4_ [all Sigma], and protease inhibitors [Complete Protease Inhibitor Cocktail, Roche]) on ice. Confluent cultured cells were washed with ice-cold PBS twice and then lysed for 30 min in protein lysis buffer on ice. After 30 seconds of sonication (Branson Sonifier 250, intensity 4 of 10) on ice, samples were pelleted (10 min, 4°C, 15,000 RPM, Eppendorf table centrifuge) and protein containing supernatant stored at −80°C. A total of 15µg of total protein was used for Western blotting. Protein samples were boiled (5 min, 95°C) and separated by polyacrylamide gel electrophoresis using 4–15% gradient gels (BioRad) followed by transfer onto nitrocellulose membranes according to the supplier's protocol (Mini ProteanIII, BioRad). Membranes were blocked in PBS, 0.1% Tween20 (PBS-T, Sigma) +5% dry milk powder (BioRad). Incubation times for primary and secondary antibodies were 2 hours and 1 hour at room temperature, respectively. Antibodies were diluted in PBS-T +2% dry-milk powder. Membranes were washed 5 times in PBS-T between antibody incubations, and films were developed using ECL plus (Amersham).

### Immunofluorescence

Cells were cultured in 8 chamber glass slides (BD Falcon), washed twice with ice-cold PBS and then fixed with methanol/acetone (1∶1 vol/vol) for 15 min at 4°C. After fixation cells were washed with PBS twice and blocked with 500µl PBS/2% dry-milk powder for 20 min at room temperature. Antibody staining was performed in 100µl PBS for 90 min at 37°C or 4°C overnight. If needed, fluorophor-labeled secondary antibodies directed against the appropriate host, were applied for 45 min at room temperature after 3 washes with PBS. After a further 3 washes with PBS, glass slides were mounted using VECTASHIELD with DAPI (Vector Labs). All immunofluorescence pictures were taken with a Leica DM1000 microscope featuring a Leica DFC FX camera (Leica Microsystems). Tumor sections of patient material or xenografts were snap frozen embedded in OCT compound (Tissue-Tek) on dry ice. OCT embedded tissues were then stored at −80°C and equilibrated to −20°C for at least 1 hour prior to sectioning. Tumor tissue was sliced (8 microns) using a Leica CM 1850 cryostat (Leica Microsystems) and then transferred onto Superfrost slides (Fisher Scientific). Slides were fixed in acetone (Fisher Scientific) for 10 min at −20°C. After two rinses with PBS (Sigma) slides were blocked with 2% milk powder (BioRad) in PBS for 20 min at room temperature.

### Antibodies

All antibodies and the dilutions used in this study are detailed in Table S2.

## Supporting Information

Figure S1 **Transdifferentiation of clonal epithelial cells into mesenchymal cells.** **A**) Epithelial clone derived from ovc316-XC at passage 8 and passage 20 stained for the epithelial markers E-cadherin and Claudin 7. **B**) Passage 20 cells stained for the mesenchymal marker N-p120. The scale bar is 40µm.
(PDF)

Figure S2 **Characterization of E/M hybrid cells.** **A**) E-cadherin^high^ cells contain low amounts of CD31, caldesmon, and VCAM-1, whereas E-cadherin^low^ cells stain highly positive for these markers. **B**) Analysis of pluripotency markers. E-cadherin^low^ cells express high levels of nuclear-localized Nanog and Oct4. Most cells in ovc316 cultures contain high amounts of nuclear Sox2. **C**) Correlation of membrane-bound beta-catenin (green) and claudin 7 (red). Cells low on membrane-claudin 7 predominantly localize beta-catenin to the cytoplasm/nucleus. Shown are images from ovc316-XC. D) Staining for human mitochondrial marker. Immunofluorescence analysis of ovc0117-PC, ovc0122-PC, ovc100506-XC, and ovc100728-XC revealed similar results.
(PDF)

Figure S3 **Characterization of different cellular phenotypes ovarian cancer cultures.** **A**) Analysis of E-cadherin (x axis), CD44 (y axis), and CD133 (histogram). Shown are density blots of the whole culture (left panel) and of the CD133^+^ sub-fraction within the culture (right panel). CD133^+^ cancer stem-like cells are highly positive for CD44 and are divided into epithelial and mesenchymal phenotypes. The mesenchymal sub-fraction enriches during passaging and concomitant EMT in culture ovc316. The total amount of CD133 dramatically decreases after passaging. Shown are studies with ovc316-XC. **B**) Analysis of E-cadherin (x axis), CD44 (y axis), and Tie2 (histogram). Shown are density blots of the whole culture (left panel) and of the Tie2^+^ sub-fraction within the culture (right panel). Tie2^+^ progenitor cells have an epithelial phenotype and are lower in CD44 levels than CD133^+^ cells. Shown are studies with ovc316-XC. **C**) Flow cytometry analysis of clonal ovc316-XC cultures. Non-tumorigenic epithelial and mesenchymal clones are low on CD133. Tumorigenic E/M hybrid cultures contain CD133^+^ cells of epithelial and mesenchymal phenotypes that are highly positive for CD44. **D**) Sphere formation after growth factor starvation in primary ovarian cancer cultures from different biopsies. Primary cultures were grown In MEBM medium for 5 days. Shown are studies with four primary cultures.
(PDF)

Figure S4 **E/M cells subsets in biopsies from ovarian cancer patients.** **A**) Sections from patient biopsies were stained for the epithelial marker E-cadherin and the mesenchymal marker Vimentin. **B and C**) Sections from patient biopsies were stained for E-cadherin and CD133 (B) or E-cadherin and Tie2 (C). CD133^+^ areas with differential E-cadherin staining are marked. Shown are sections of ovc1208-biopsy, ovc0122 biopsy, and ovc0117-biopsy. Immunofluoresence analysis of ovc1123-biopsy, ovc0111-biopsy, ovc0116-biopsy, ovc123-biopsy, and ovc100506-biopsy resulted in similar staining.
(PDF)

Figure S5 **Sections of ovc31-X xenografts.** **A**) Global view of a xenograft tumor assembled from different images. **B**) Higher magnification of an ovc316-X xenograft section to visualize two different CD133^+^ subsets, including areas with membrane E-cadherin and areas with punctated cytoplasmic E-cadherin.
(PDF)

Figure S6 **Analysis of CD133 fractions.** **A**) Sections of tumors derived from sorted CD133^+^ and CD133^−^ cells. **B**) Flow cytometry analysis for differentiation and proliferation markers. CD133^+^ and CD133^−^ cells within ovc316-X xenograft tumors are positive for Ki-67. CD133^−^ cells contain higher amounts of Ki-67 negative cells than the CD133^+^ cell fraction.
(PDF)

Figure S7 **Ovarian cancer cell lines contain CD133^+^ and Tie2^+^ cells.** The majority of CD133^+^ and Tie2^+^ cells are mutually exclusive. Tie2^+^/CD133^+^ double positive cells have lower levels of CD133^+^ than CD133^+^/Tie2^−^ cells. **A**) SKOV3-ip1 cells. **B**) OVCAR-5 cells. **C**) OVCAR-3 cells
(PDF)

Figure S8 **Expression of E-cadherin and stem cell markers CD133 and Nanog after growth factor/serum starvation of passage 18 ovc316-XC cells.**
(PDF)

Table S1 (PDF)

Table S2 (PDF)
