# Correction: Analysis of Epithelial and Mesenchymal Markers in Ovarian Cancer Reveals Phenotypic Heterogeneity and Plasticity

**DOI:** 10.1371/annotation/8c637352-3614-406c-89dc-e78d10fa069c

**Published:** 2011-02-11

**Authors:** Robert Strauss, Zong-Yi Li, Ying Liu, Ines Beyer, Jonas Persson, Pavel Sova, Thomas Möller, Sari Pesonen, Akseli Hemminki, Petra Hamerlik, Charles Drescher, Nicole Urban, Jiri Bartek, André Lieber

The authors would like to add the following to the funding information: "The Danish National Research Foundation, the Danish Cancer Society, the European Commission (projects Biomedreg-CZ.1.05/2.1.00/01.0030 and Infla-Care), and the Lundbeck Foundation (R13-A1287).

