# Supplementary figures and images for: Analysis of Epithelial and Mesenchymal Markers in Ovarian Cancer Reveals Phenotypic Heterogeneity and Plasticity
Source: PLoS One. 2011 Jan 14;6(1):e16186. doi: 10.1371/journal.pone.0016186 (PMC3021543; doi:10.1371/journal.pone.0016186)

**A**

E-cadherin

Claudin 7

Passage 8

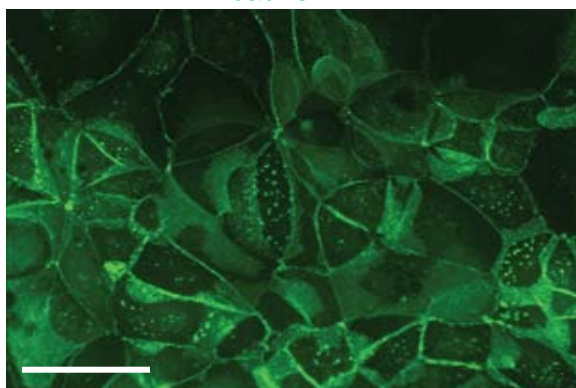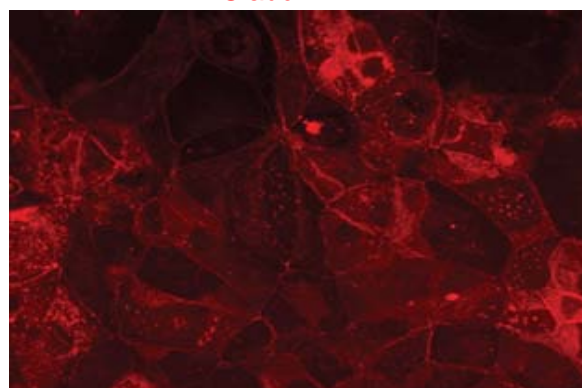

Passage 20

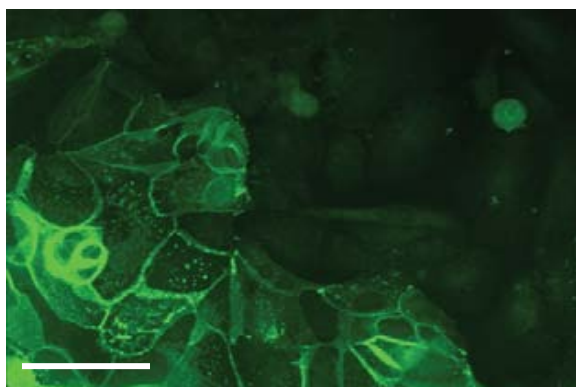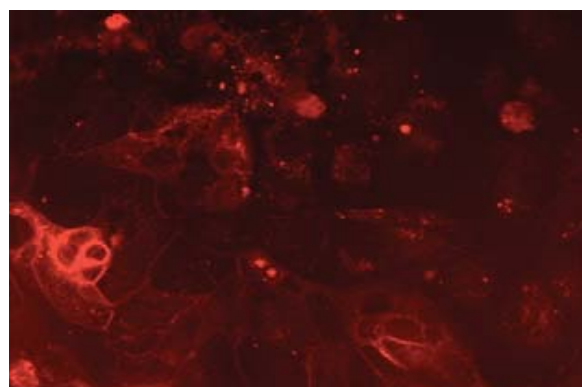**B**

E-cadherin

N-p120 catenin

Passage 20

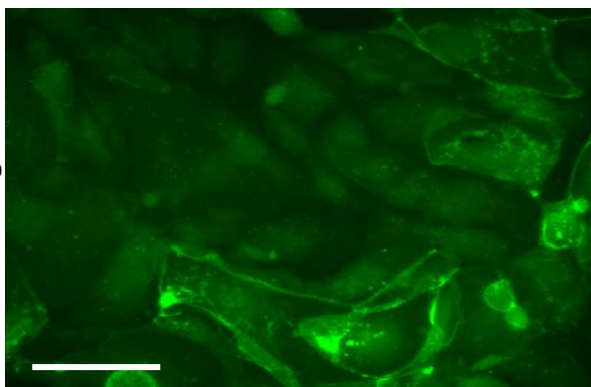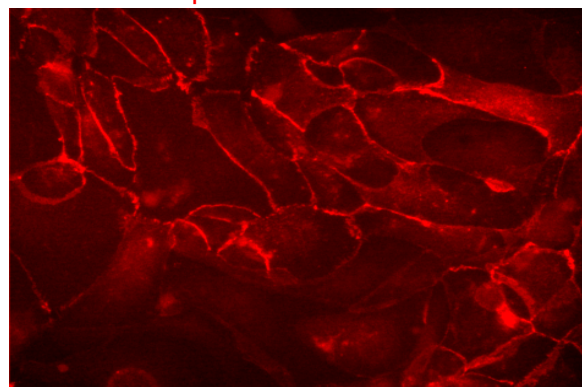**Figure S1**

Supplement: Figure S1 — Transdifferentiation of clonal epithelial cells into mesenchymal cells. A) Epithelial clone derived from ovc316-XC at passage 8 and passage 20 stained for the epithelial markers E-cadherin and Claudin 7. B) Passage 20 cells stained for the mesenchymal marker N-p120. The scale bar is 40µm. (PDF) [file pone.0016186.s001.pdf]

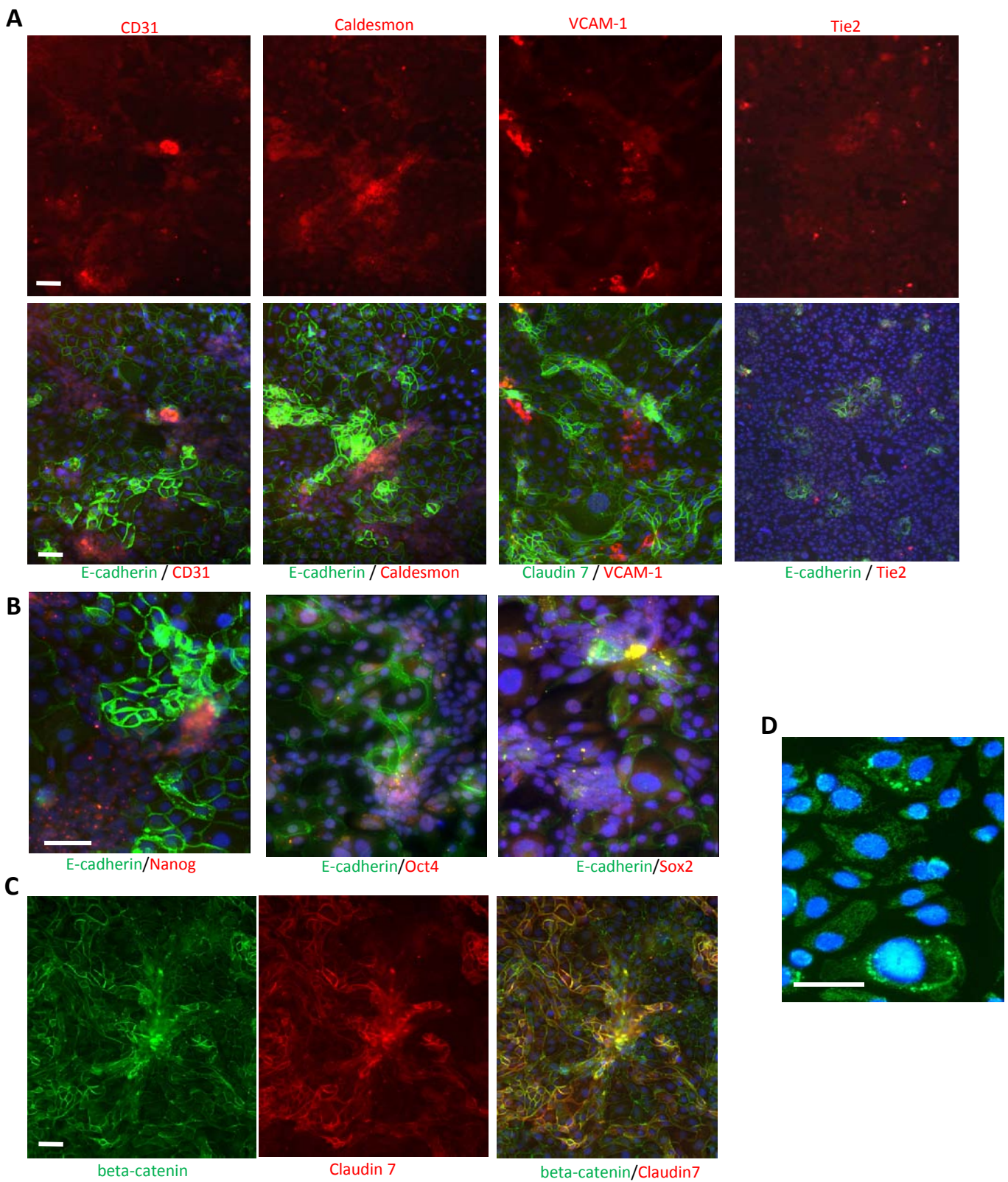

**Figure S2**

Supplement: Figure S2 — Characterization of E/M hybrid cells. A) E-cadherinhigh cells contain low amounts of CD31, caldesmon, and VCAM-1, whereas E-cadherinlow cells stain highly positive for these markers. B) Analysis of pluripotency markers. E-cadherinlow cells express high levels of nuclear-localized Nanog and Oct4. Most cells in ovc316 cultures contain high amounts of nuclear Sox2. C) Correlation of membrane-bound beta-catenin (green) and claudin 7 (red). Cells low on membrane-claudin 7 predominantly localize beta-catenin to the cytoplasm/nucleus. Shown are images from ovc316-XC. D) Staining for human mitochondrial marker. Immunofluorescence analysis of ovc0117-PC, ovc0122-PC, ovc100506-XC, and ovc100728-XC revealed similar results. (PDF) [file pone.0016186.s002.pdf]

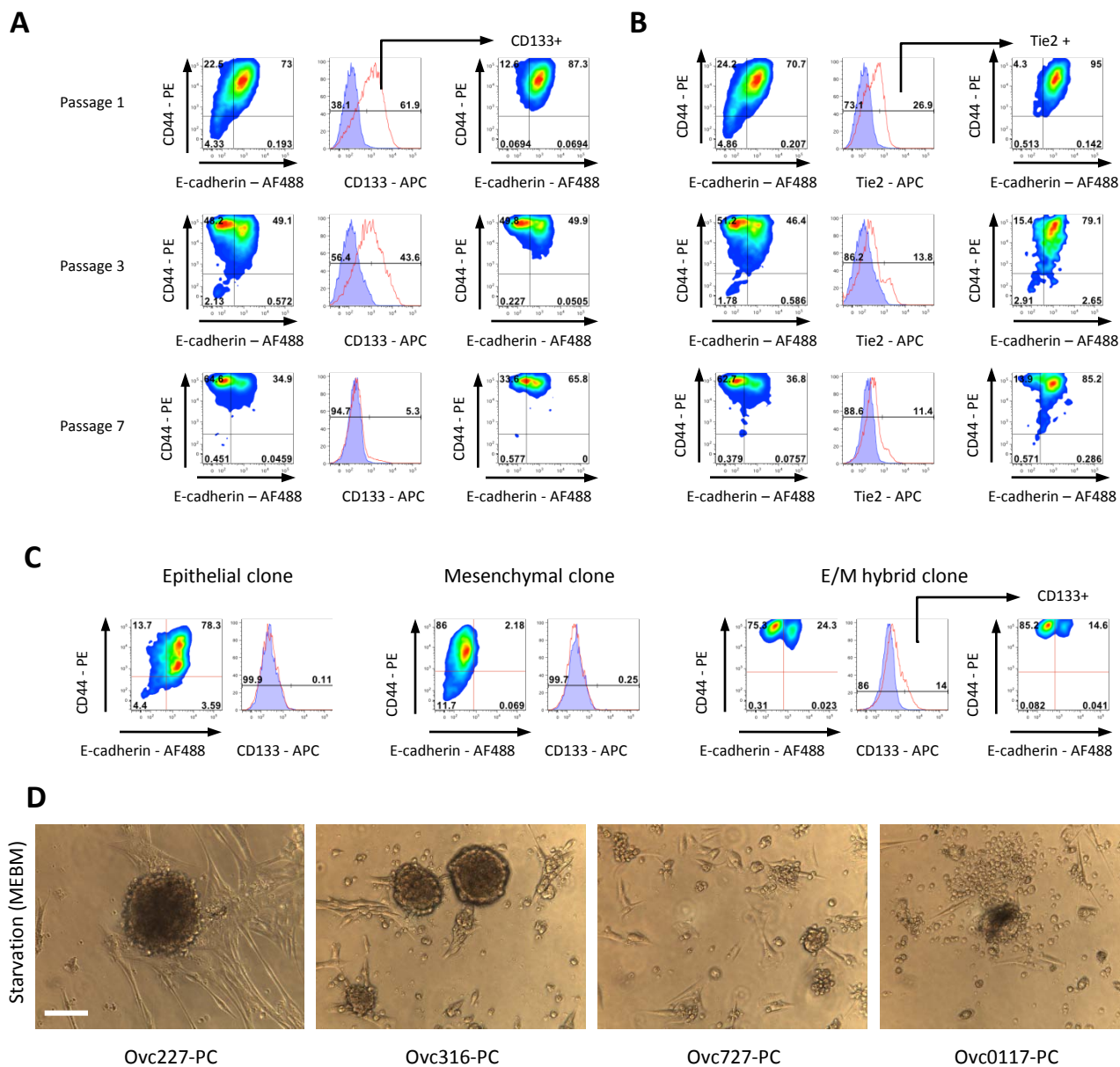

**Figure S3**

Supplement: Figure S3 — Characterization of different cellular phenotypes ovarian cancer cultures. A) Analysis of E-cadherin (x axis), CD44 (y axis), and CD133 (histogram). Shown are density blots of the whole culture (left panel) and of the CD133+ sub-fraction within the culture (right panel). CD133+ cancer stem-like cells are highly positive for CD44 and are divided into epithelial and mesenchymal phenotypes. The mesenchymal sub-fraction enriches during passaging and concomitant EMT in culture ovc316. The total amount of CD133 dramatically decreases after passaging. Shown are studies with ovc316-XC. B) Analysis of E-cadherin (x axis), CD44 (y axis), and Tie2 (histogram). Shown are density blots of the whole culture (left panel) and of the Tie2+ sub-fraction within the culture (right panel). Tie2+ progenitor cells have an epithelial phenotype and are lower in CD44 levels than CD133+ cells. Shown are studies with ovc316-XC. C) Flow cytometry analysis of clonal ovc316-XC cultures. Non-tumorigenic epithelial and mesenchymal clones are low on CD133. Tumorigenic E/M hybrid cultures contain CD133+ cells of epithelial and mesenchymal phenotypes that are highly positive for CD44. D) Sphere formation after growth factor starvation in primary ovarian cancer cultures from different biopsies. Primary cultures were grown In MEBM medium for 5 days. Shown are studies with four primary cultures. (PDF) [file pone.0016186.s003.pdf]

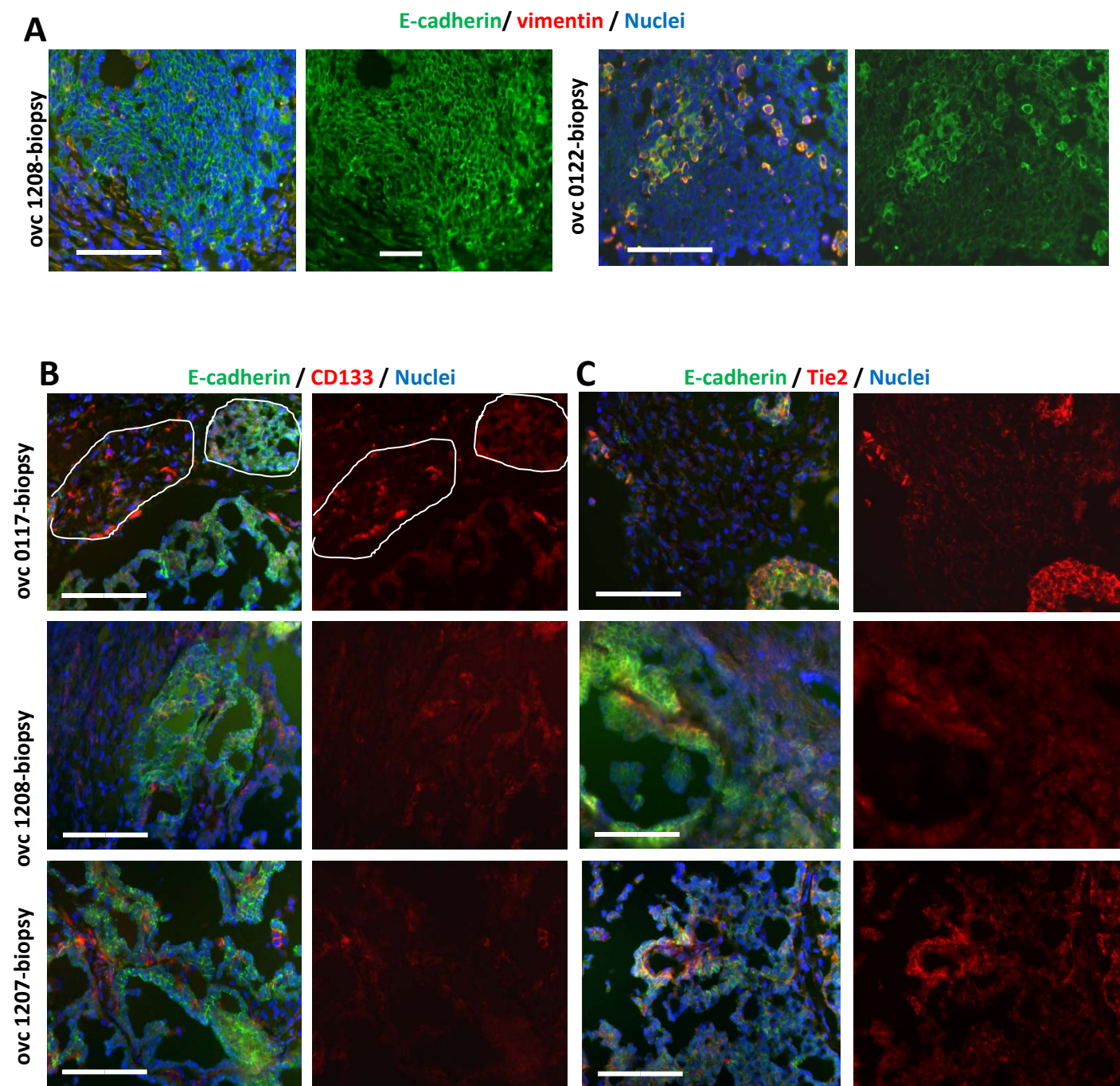

**Figure S4**

Supplement: Figure S4 — E/M cells subsets in biopsies from ovarian cancer patients. A) Sections from patient biopsies were stained for the epithelial marker E-cadherin and the mesenchymal marker Vimentin. B and C) Sections from patient biopsies were stained for E-cadherin and CD133 (B) or E-cadherin and Tie2 (C). CD133+ areas with differential E-cadherin staining are marked. Shown are sections of ovc1208-biopsy, ovc0122 biopsy, and ovc0117-biopsy. Immunofluoresence analysis of ovc1123-biopsy, ovc0111-biopsy, ovc0116-biopsy, ovc123-biopsy, and ovc100506-biopsy resulted in similar staining. (PDF) [file pone.0016186.s004.pdf]

**A**

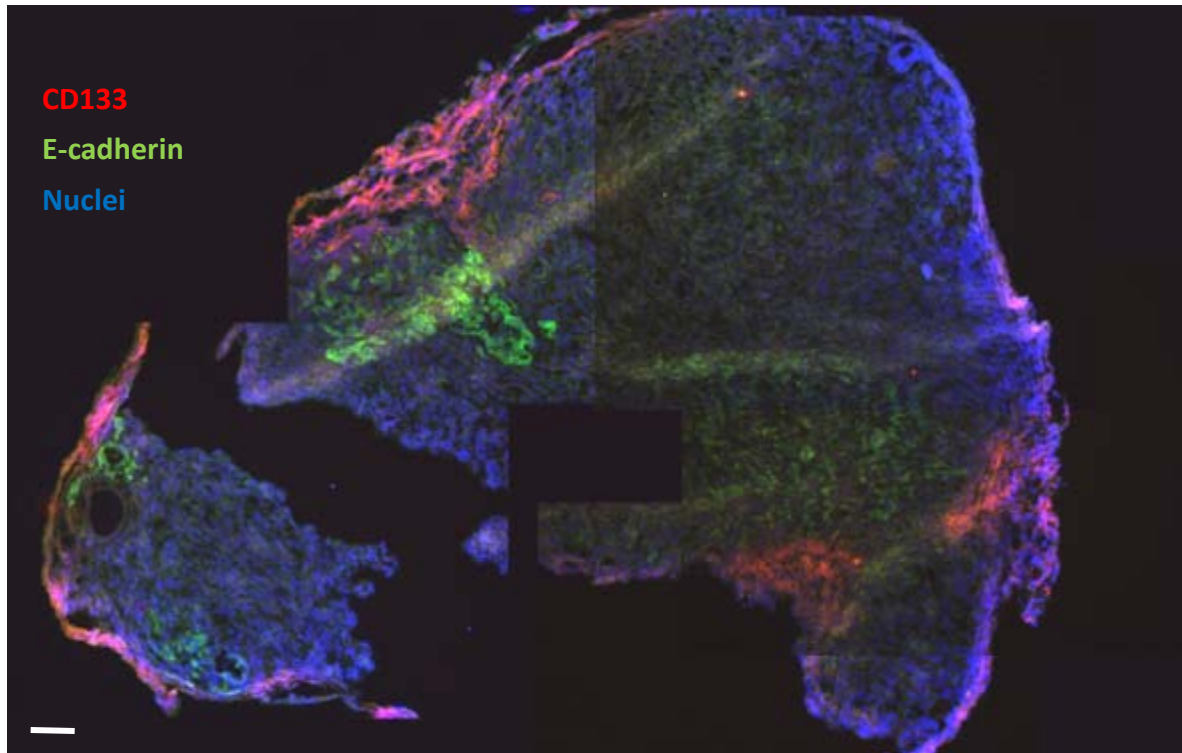

**B**

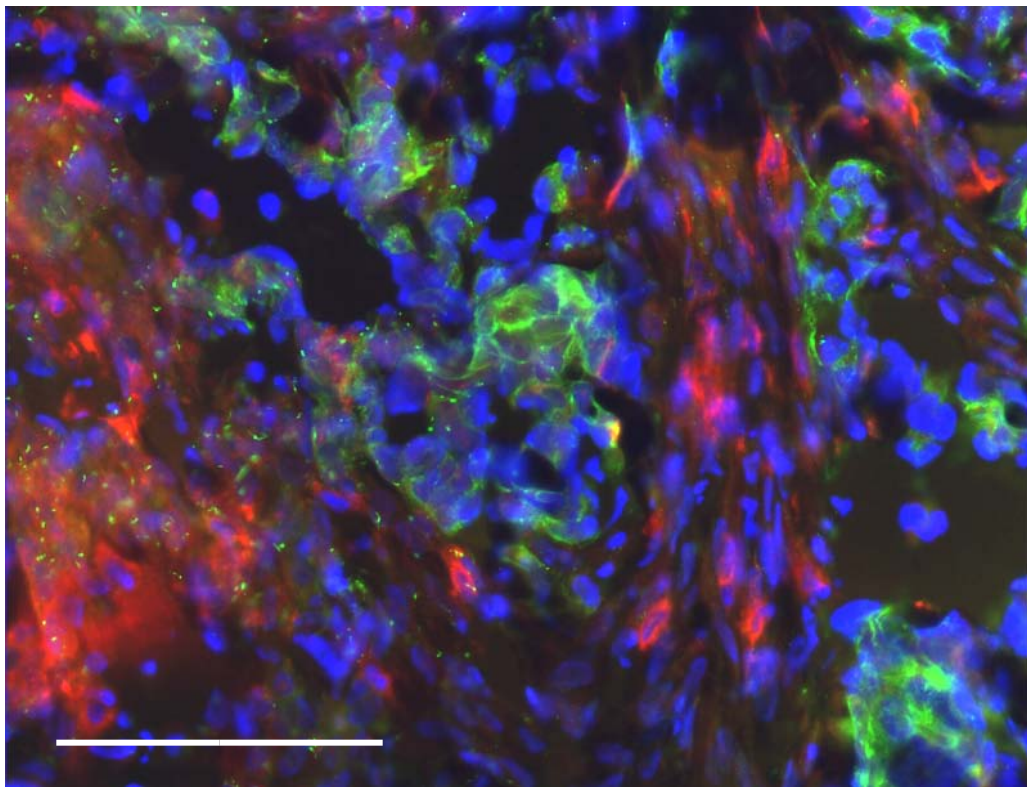

**Figure S5**

Supplement: Figure S5 — Sections of ovc31-X xenografts. A) Global view of a xenograft tumor assembled from different images. B) Higher magnification of an ovc316-X xenograft section to visualize two different CD133+ subsets, including areas with membrane E-cadherin and areas with punctated cytoplasmic E-cadherin. (PDF) [file pone.0016186.s005.pdf]

**A**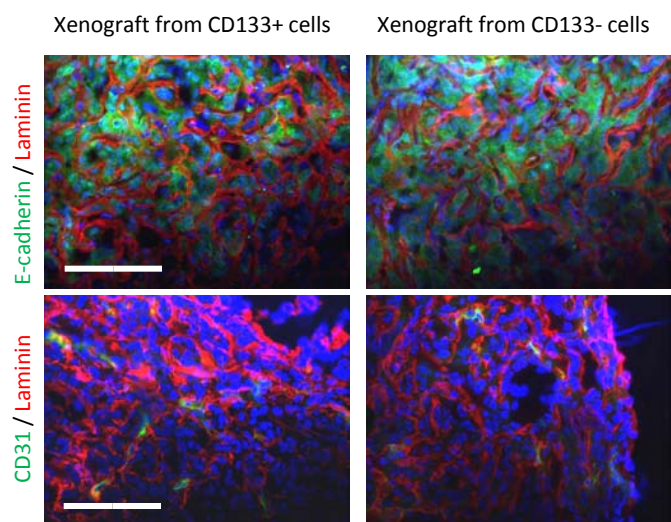**B**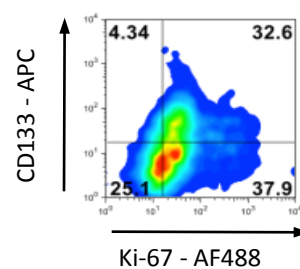**Figure S6**

Supplement: Figure S6 — Analysis of CD133 fractions. A) Sections of tumors derived from sorted CD133+ and CD133− cells. B) Flow cytometry analysis for differentiation and proliferation markers. CD133+ and CD133− cells within ovc316-X xenograft tumors are positive for Ki-67. CD133− cells contain higher amounts of Ki-67 negative cells than the CD133+ cell fraction. (PDF) [file pone.0016186.s006.pdf]

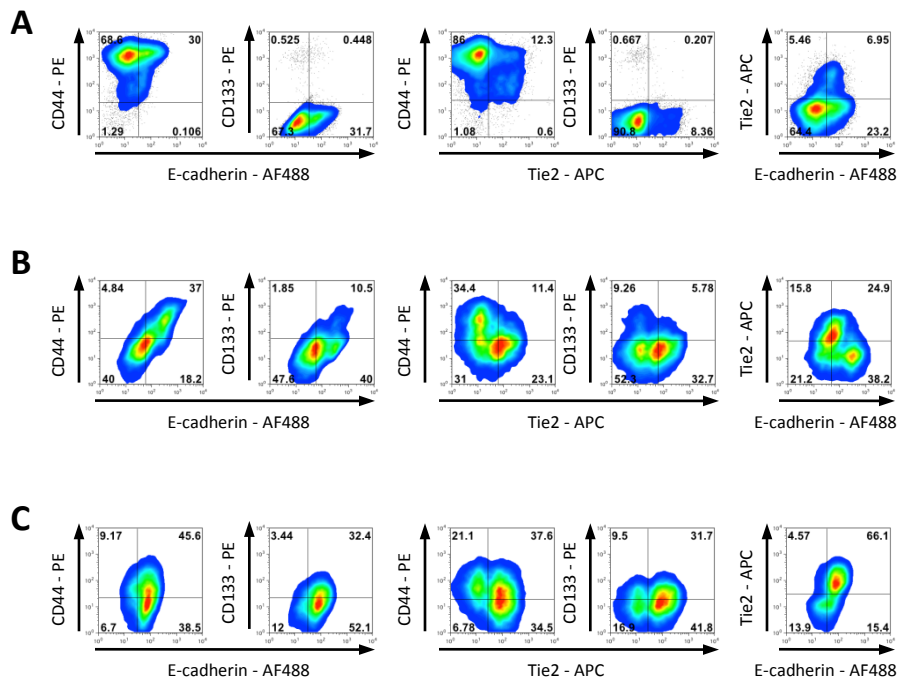

**Figure S7**

Supplement: Figure S7 — Ovarian cancer cell lines contain CD133+ and Tie2+ cells. The majority of CD133+ and Tie2+ cells are mutually exclusive. Tie2+/CD133+ double positive cells have lower levels of CD133+ than CD133+/Tie2− cells. A) SKOV3-ip1 cells. B) OVCAR-5 cells. C) OVCAR-3 cells (PDF) [file pone.0016186.s007.pdf]

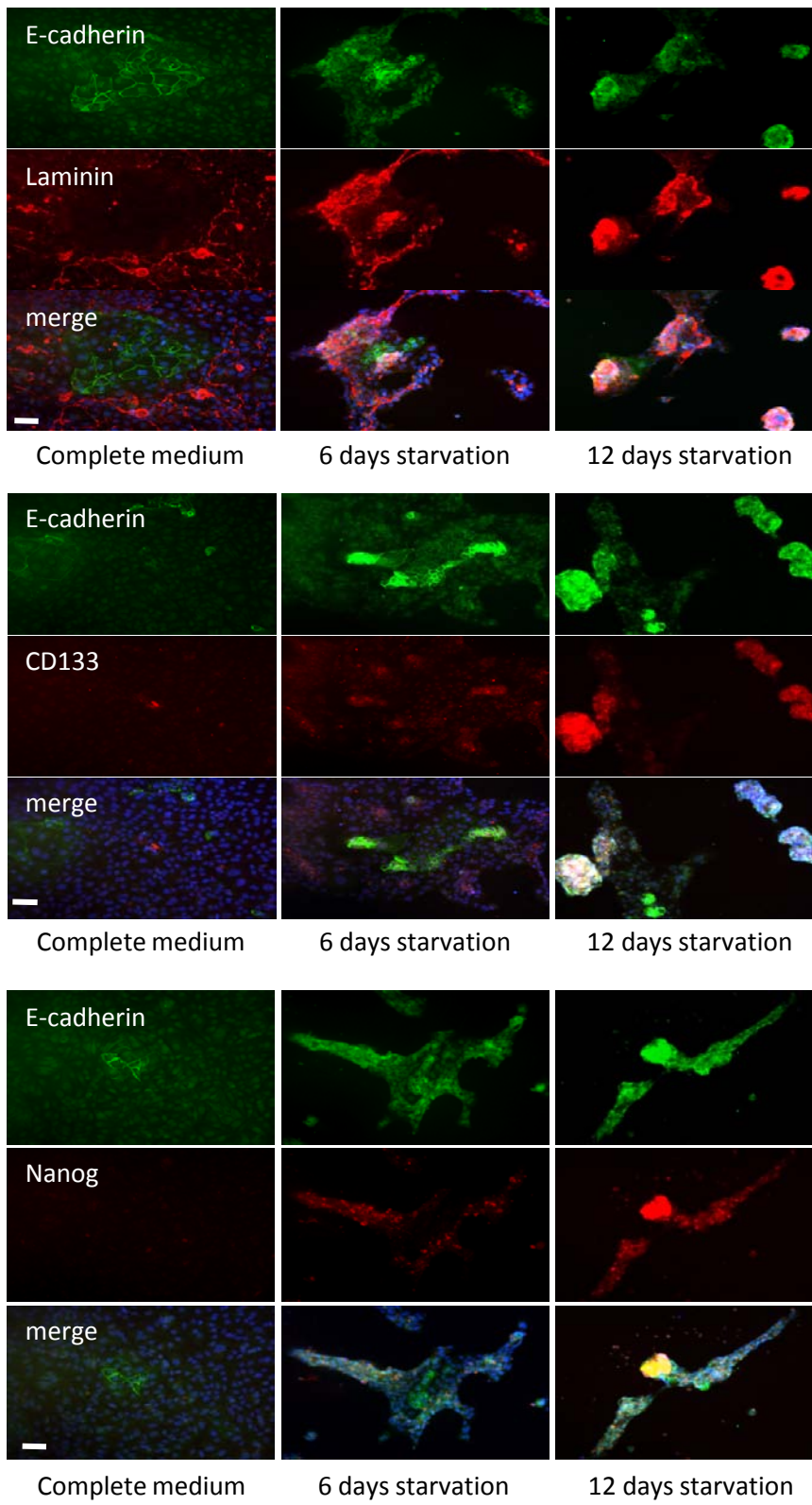

**Figure S8**

Supplement: Figure S8 — Expression of E-cadherin and stem cell markers CD133 and Nanog after growth factor/serum starvation of passage 18 ovc316-XC cells. (PDF) [file pone.0016186.s008.pdf]
