# Supplementary material for: Analysis of Epithelial and Mesenchymal Markers in Ovarian Cancer Reveals Phenotypic Heterogeneity and Plasticity
Source: PLoS One. 2011 Jan 14;6(1):e16186. doi: 10.1371/journal.pone.0016186 (PMC3021543; doi:10.1371/journal.pone.0016186)
Supplement: Table S1 — (PDF) [file pone.0016186.s009.pdf]

Table S1

primary ovarian cancer cultures that were passageable >10x					
	**origin**	**chemotherapy**	**sphere formation**	**tumorigenic**	**CD133+ E/M cells *in vivo*****
He 1070	biopsy		x	x	x
He 109M	biopsy		x	x	x
He 122	biopsy		x	x	x
He 145	biopsy		x	x	x
ovc 0117	biopsy		x		n.t.
ovc 0117-2	ascites*			n.t.	n.t.
ovc 0122	biopsy		x		x
ovc 1204	ascites				n.t.
ovc 1207	biopsy				x
ovc 1208	biopsy		x		x
ovc 1276	ascites				n.t.
ovc 227	biopsy		x		x
ovc 316	biopsy	x	x	x	x
ovc 316om	biopsy/met	x	x	x	x
ovc 405	biopsy	x	x	x	x
ovc 514	biopsy		x		x
ovc 514-2	ascites*				n.t.
ovc 615	biopsy				n.t.
ovc 622	biopsy				n.t.
ovc 624	biopsy	x			n.t.
ovc 712	biopsy				n.t.
ovc 727	biopsy		x		x
ovc 727-2	ascites*			n.t.	n.t.
ovc 910	biopsy		x		x

primary ovarian cancer cultures that were passageable <10x		
	**origin**	**chemotherapy**
ovc 0111	biopsy	x
ovc 0112	biopsy	
ovc 0112-2	ascites*	
ovc 0119	biopsy	x
ovc 0120	biopsy	
ovc 0121	biopsy	
ovc 1025	biopsy	
ovc 1102	biopsy	
ovc 1102-2	ascites*	
ovc 1105	biopsy	
ovc 1122	ascites	
ovc 1123	biopsy	
ovc 118	biopsy	
ovc 12032	ascites	
ovc 210	biopsy	x
ovc 608	biopsy	
ovc 609	biopsy	x
ovc 708	biopsy	
ovc 921	biopsy	
ovc 922	biopsy	

Nomenclature

n.t. - not tested

* - matching ascites to biopsy from same patient

**-immunofluoresence analysis of biopsy sections
