# Supplementary material for: Analysis of Epithelial and Mesenchymal Markers in Ovarian Cancer Reveals Phenotypic Heterogeneity and Plasticity
Source: PLoS One. 2011 Jan 14;6(1):e16186. doi: 10.1371/journal.pone.0016186 (PMC3021543; doi:10.1371/journal.pone.0016186)
Supplement: Table S2 — (PDF) [file pone.0016186.s010.pdf]

Table S2

Antibody	Host	Dilution	Vendor
**For immunohistochemistry**			
b-catenin-FITC	mouse	1:100	BD Transduction Laboratories, San Jose, CA,
AE1/AE3	mouse	1:100	BD Transduction Laboratories, San Jose, CA,
CA125	mouse	1:100	Dako, Glostrup, Denmark
Caldesmon	rabbit	1:100	abcam, Cambridge, MA
CD133	rabbit	1:500	abcam
CD31	rabbit	1:100	abcam
CEA	rabbit	1:100	Biomeda Corp, Foster City, CA
Claudin7	rabbit	1:100	abcam
E-cadherin	mouse	1:200	Sigma, St Louis MO
E-cadherin	rabbit	1:100	Cell Signaling Technology, Inc, Danvers, MA
E-cadherin-FITC	mouse	1:200	BD Biosciences, San Jose, CA
EpCAM	mouse	1:100	Biomeda Corp
human mitochondria	mouse	1:200	BD Transduction Laboratories, San Jose, CA,
Laminin	rabbit	1:400	Dako, Glostrup, Denmark
N-cadherin	rabbit	1:150	GeneTex Inc, Miaoli, Taiwan, R.O.C
Nanog	mouse	1:10?	R&D System , Minneapolis, MN
NGAL	rabbit	1:100	abcam
Oct-4	rabbit	1:200	Cell Signaling Technology, Inc, Danvers, MA
Snail	rabbit	1:100	abcam
Sox2	rabbit	1:100	abcam
Tie 2	goat	1:100	R&D System
Twist	rabbit	1:100	abcam
VCAM1	mouse	1:100	abcam
Vimentin	mouse	1:200	Sigma,
Vimentin	rabbit	1:100	Sigma,
Anti-rabbit-AF488	goat	1:400	Invitrogen/ Molecular Probes, Eugene, OR
Anti-rabbit-AF568	goat	1:400	Invitrogen/ Molecular Probes
Anti-mouse-AF488	goat	1:400	Invitrogen/ Molecular Probes
Anti-mouse-AF568	goat	1:400	Invitrogen/ Molecular Probes
Anti-goat-AF568	donkey	1:400	Invitrogen/ Molecular Probes
Anti-rat-AF568	goat	1:400	Invitrogen/ Molecular Probes
Anti-rabbit-AF405	goat	1:200	Invitrogen/ Molecular Probes
**For flow cytometry**			
CD133/1-APC	mouse	1:10	Miltenyi Biotec, Auburn, CA
CD133/1-PE	mouse	1:10	Miltenyi Biotec
CD44-PE	mouse	1:10	BD Pharmingen, San Diego, CA
E-cadherin-AF488	mouse	1:20	Biolegend, San Diego, CA
EpCAM-FITC	mouse	1:10	Stem Cell Technologies Inc, Vancouver, BC, Canada
Ki-67	rabbit	1:10	Stem Cell Technologies Inc, Vancouver, BC, Canada
Tie2-APC	mouse	1:10	R&D System
Vimentin-PE	mouse	1:10	abcam
Anti-rabbit-AF488	goat	1:200	Invitrogen/ Molecular Probes
**For MACS and FACS sorting**			
Anti-biotin MicroBeads			Miltenyi Biotec
CD133/2-biotin	mouse	1:5	Miltenyi Biotec
E-cadherin-APC	mouse	1:10	Biolegend Inc, San Diego, CA
Tie2-PE	mouse	1:5	R&D System
**For Western blotting**			
CD133	mouse	1:500	Miltenyi Biotec
E-cadherin	rabbit	1:1000	Cell Signaling Technology, Inc
GAPDH	mouse	1:1000	abcam
p38 MAPK	mouse	1:1000	Cell Signaling Technology, Inc
p44/42 MAPK (Erk1/2)	mouse	1:1000	Cell Signaling Technology, Inc
Pan-Ras	mouse	1:2000	EMD Calbiochem, Gibbstown, NJ
Phospho-cRaf	rabbit	1:1000	Cell Signaling Technology, Inc
